# Hippocampal γCaMKII dopaminylation promotes synaptic-to-nuclear signaling and memory formation

**DOI:** 10.1101/2024.09.19.613951

**Published:** 2024-09-24

**Authors:** Andrew F. Stewart, Sasha L. Fulton, Romain Durand-de Cuttoli, Robert E. Thompson, Peng-Jen Chen, Elizabeth Brindley, Bulent Cetin, Lorna A. Farrelly, Rita Futamura, Sarah Claypool, Ryan M. Bastle, Giuseppina Di Salvo, Christopher Peralta, Henrik Molina, Erdene Baljinnyam, Samuele G. Marro, Scott J. Russo, Robert J. DeVita, Tom W. Muir, Ian Maze

**Affiliations:** 1 Nash Family Department of Neuroscience, Friedman Brain Institute, Icahn School of Medicine at Mount Sinai, New York, New York 10029, USA; 2 Department of Chemistry, Princeton, New Jersey 08544, USA; 3 Department of Pharmacological Sciences and Drug Discovery Institute, Icahn School of Medicine at Mount Sinai, New York, New York 10029, USA; 4 Department of Psychiatry and Neuropsychology, School for Mental Health and Neuroscience (MHeNs), Maastricht University, Maastricht, The Netherlands; 5 The Rockefeller University Proteomics Resource Center, The Rockefeller University, New York, NY 10065, USA; 6 Institute for Regenerative Medicine, Icahn School of Medicine at Mount Sinai, New York, New York 10029, USA; 7 Drug Discovery Institute, Icahn School of Medicine at Mount Sinai, New York, New York 10029, USA; 8 Howard Hughes Medical Institute, Icahn School of Medicine at Mount Sinai, New York, New York 10029, USA

## Abstract

Protein monoaminylation is a class of posttranslational modification (PTM) that contributes to transcription, physiology and behavior. While recent analyses have focused on histones as critical substrates of monoaminylation, the broader repertoire of monoaminylated proteins in brain remains unclear. Here, we report the development/implementation of a chemical probe for the bioorthogonal labeling, enrichment and proteomics-based detection of dopaminylated proteins in brain. We identified 1,557 dopaminylated proteins – many synaptic – including γCaMKII, which mediates Ca^2+^-dependent cellular signaling and hippocampal-dependent memory. We found that γCaMKII dopaminylation is largely synaptic and mediates synaptic-to-nuclear signaling, neuronal gene expression and intrinsic excitability, and contextual memory. These results indicate a critical role for synaptic dopaminylation in adaptive brain plasticity, and may suggest roles for these phenomena in pathologies associated with altered monoaminergic signaling.

Biogenic amines, such as serotonin, dopamine and histamine, are a large class of evolutionarily ancient signaling molecules. These metabolites are found in diverse tissues in animals and can elicit biological effects in an auto-, para- or endocrine manner (1, 2). In brain, these monoamines are thought to function primarily via synaptic release, orchestrating diverse behaviors, including mood, appetite, pleasure, and sleep, as well as mediating cognitive functions, such as attention, learning, and memory (3–5). Altered monoaminergic signaling is linked to many mental health/neurological disorders, including depression and addiction, and in the case of dopamine, Parkinson’s disease (6). The canonical signaling pathways activated by biogenic amines are reasonably well understood (7–9) – they engage cognate cell surface receptors, typically G protein coupled receptors (GPCRs), of which there can be several members for each monoamine type, leading to activation of intracellular signaling cascades that elicit a number of cellular outputs (10). While these pathways rely on vesicular packaging and synaptic release of monoamines, it has long been recognized that non-vesicular pools of monoamines (both cytoplasmic and nuclear) exist in various cell-types, including neurons (11, 12). The existence of these pools suggested a non-canonical, receptor-independent role for monoamines in cell biology. Evidence for this goes back several decades, where it was found that biogenic amines (both mono- and polyamines) can be covalently attached to proteins by transglutaminase enzymes (13–15).

Motivated by these and other more recent findings (16–18), we previously asked whether this class of posttranslational modification (PTM) can occur on histones, the core packing proteins of chromatin. Tissue transglutaminase 2 (TGM2)-mediated serotonylation (19–24), dopaminylation (25–27) and histaminylation (28) of histone H3 have all now been documented and are found in both monoaminergic and non-monoaminergic cells of the brain. The genomic enrichment patterns and overall dynamics of these histone monoaminylations argue for critical roles in transcriptional, physiological and behavioral plasticity as part of both normal neural development, and in the context of brain pathologies. However, given the enzymatic promiscuity of TGM2, it seemed possible – perhaps even likely based upon our previous data – that glutamine-containing proteins in other cellular compartments in brain may also be subject to monoaminylation. However, the full extent of protein monoaminylations across neural tissues remains unknown. By extension, it is also unclear how the levels or prevalence of these modifications may change as a function of cell/tissue state, whether that be through differentiation, activation of signaling pathways or environmental perturbations. As such, in this study we sought to develop an approach that would allow for the identification of the broader repertoire of monoaminylated proteins in brain (focusing here on protein dopaminylation) to more fully understand the contributions of these non-canonical monoamine signaling moieties in brain health and disease.

## Design of a chemical probe for the bioorthogonal labeling, enrichment and detection of dopaminylated proteins

Inspired by earlier work (29, 30) – and in agreement with recent *in vitro* findings (31, 32) – demonstrating that 1,2 catechols (e.g., dopamine/DA, norepinephrine/NE) can undergo a strain-promoted cycloaddition reaction with cyclooctynes following oxidation to orthoquinones, we developed a modified bioorthogonal strategy whereby periodate treated catecholaminylated peptides can be efficiently labeled with a biotinylated strained cyclooctyne probe (Bio-CO) (Fig. S2A); see Fig. S1A–C for synthesis characterization. First, to validate that a known substrate of protein dopaminylation can be efficiently IP’d from neural tissues, Bio-CO labeling and enrichment (using Streptavidin as prey) of histone H3 from mouse brain extracts was performed, which confirmed that catecholaminylated H3 was robustly labeled by the probe (+ probe *vs*. − probe comparisons are provided to demonstrate a lack of background signal in the absence of probe labeling) (Fig. S2B). To then assess the specificity of our probe against different monoaminylated substrates, we performed *in vitro* transglutaminase reactions against recombinant histone H3 using various monoamine donors (serotonin/5-HT, histamine/His, DA and NE), followed by Bio-CO enrichment and western blotting for H3. Importantly, since H3 can be modified by multiple catecholamines (DA and NE; see Fig. 2C–F for *in vitro* validations of H3Q5 noradrenylation) in a TGM2-dependent manner, we wished to explore whether the Bio-CO probe equally labels a dopaminylated *vs*. noradrenylated substrate. We found that Bio-CO efficiently immunoprecipitated (IP’d) dopaminylated H3, with no labeling observed for other monoaminylated forms of the protein, including noradrenylated H3 (Fig. S2G). While, in theory, Bio-CO would also be predicted to label noradrenylated substrates, we found that under the specific reaction conditions used in this study that no such labeling was observed, possibly owing to steric hindrance between the probe and the β-hydroxyl of NE (Fig. S2H), although the precise reasons for this lack of labeling remain unclear. While further optimization of reaction conditions may result in efficient labeling of noradrenylated proteins, we viewed such selectivity towards dopaminylated substrates as an advantage in our study. To further assess the efficacy and selectivity of the Bio-CO probe in labeling dopaminylated *vs*. noradrenylated proteins in neurons, we treated cultured primary (DIV14) mouse corticostriatal neurons (which, *in vivo*, do not synthesize the monoamines but rather receive projections from catecholaminergic neurons) with 500 nM DA or NE for 1 hr, followed by Bio-CO labeling, Streptavidin enrichment and mass spectrometry (LC-MS/MS) (Fig. S2I). Similar to our *in vitro* findings, these results indicated that Bio-CO robustly labeled dopaminylated (Fig. S2J, Data S1), but not noradrenylated (Fig. S2K, Data S2), proteins in treated neurons. 330 uniquely dopaminylated proteins were identified by LC-MS/MS in this culture system, the vast majority of which were found to be synaptic (both pre- and post-synaptic; SynGO 2024) and/or involved in synaptic regulation (GO BP 2023), and were enriched in biological pathways known to be disrupted in brain disorders (DisGeNET) associated with altered dopamine signaling – e.g., schizophrenia, intellectual disability, Parkinson’s disease, etc. (Fig. S2L).

## Characterization of the mouse brain dopaminylome *in vivo*

**Fig. 1:**
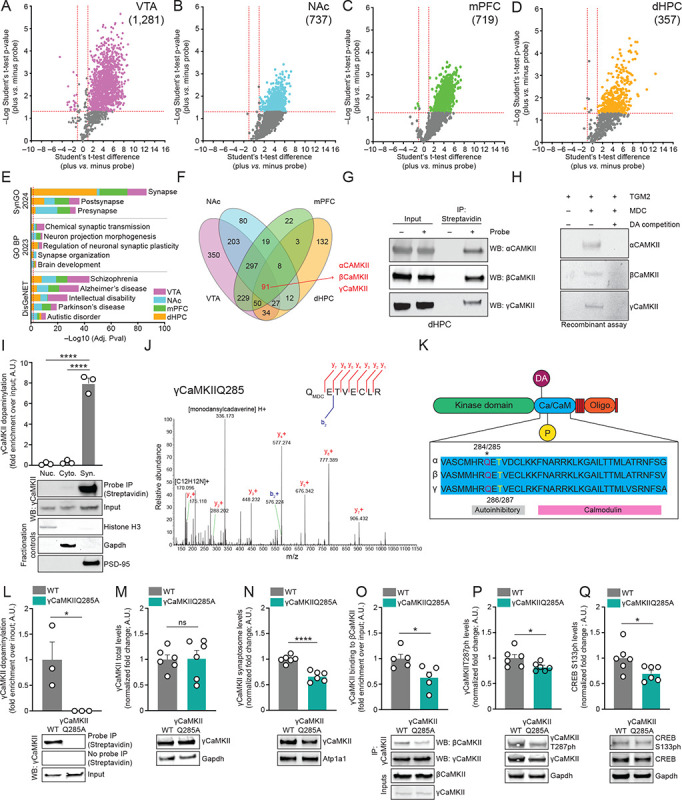
Global proteomic profiling in mammalian brain identifies roles for protein dopaminylation in synaptic-to-nuclear signaling. Volcano plots of MS-identified dopaminylated proteins enriched by the Bio-CO probe in (**A**) VTA, (**B**) NAc, (**C**) mPFC and (**D**) dHPC (Student’s t-test corrected for multiple comparisons; + probe *vs*. – probe: *n* = 3; *p*<0.05, fold-change >1). (**E**) Ontology analysis (SynGO 2024, GO BP 2023, DisGeNET) of MS-identified proteins enriched by the Bio-CO probe in VTA, NAc, mPFC and dHPC (FDR<0.05; Benjamini-Hochberg). (**F**) Venn diagram depicting the overlap of MSidentified dopaminylated proteins enriched by the Bio-CO probe in VTA *vs*. NAc *vs*. mPFC *vs*.dHPC (from **A**). (**G**) Bio-CO probe-mediated bioorthogonal labeling of endogenously dopaminylated αCaMKII, βCaMKII and γCaMKII from mouse dHPC. Inputs = 1%; Streptavidin IPs were performed for – *vs*. + probe conditions, followed by blotting for αCaMKII, βCaMKII and γCaMKII. (**H**) Bio-CO probe-mediated bioorthogonal labeling of MDCylated αCaMKII, βCaMKII and γCaMKII *in vitro* following TGM2-dependent transglutamination. DA competition revealed that MDCylation of recombinant αCaMKII, βCaMKII and γCaMKII effectively eliminates MDCylation signal for all three proteins. (**I**) Bio-CO probe-mediated bioorthogonal labeling of endogenously dopaminylated γCaMKII from sub-cellular fractions of dHPC revealed that γCaMKII dopaminylation is exclusively synaptic. Fractionation control blots for the sub-cellular compartments (H3 for nuclear, Gapdh for cytosolic and PSD-95 for synaptic) are provided, and γCaMKII levels were normalized to respective inputs (1%). *n* = 3/fraction – significance determined by one-way ANOVA (F_2,6_ = 215.9, ****p<0.0001), followed by post hoc analysis (Tukey’s MC test, ****p<0.0001). (**J**) MS spectra for MDCylation of γCaMKII at glutamine 285. Y+ and b+ ions are annotated in red and blue, respectively. (**K**) Cartoon of protein sequence alignment and domain structure for αCaMKII *vs*. βCaMKII *vs*. γCaMKII. (**L**) Bio-CO probe-mediated bioorthogonal labeling of dopaminylated γCaMKII from dHPC of wildtype *vs*. γCaMKIIQ285A mutant mice. Streptavidin IPs were performed for – *vs*. + probe conditions and γCaMKII levels were normalized to respective inputs (1%). *n* = 3/genotype – significance determined by unpaired Student’s t-test (t_4_ = 2.873, *p = 0.0453). (**M**) Western blotting for total γCaMKII levels (normalized to Gapdh as a loading control) in dHPC from wildtype *vs*. γCaMKIIQ285A mutant mice. *n* = 6/genotype – significance determined by unpaired Student’s ttest (t_10_ = 0.1185, *p* = 0.9080). (**N**) Western blotting for total γCaMKII levels in dHPC synaptic fractions (normalized to Atp1a1 as a loading control) from wildtype *vs*. γCaMKIIQ285A mutant mice. *n* = 6/genotype – significance determined by unpaired Student’s t-test (t_10_ = 6.592, ****p<0.0001). (**O**) Western blotting of dHPC co-IPs to assess βCaMKII binding to γCaMKII in wildtype *vs*. γCaMKIIQ285A mutant mice. *n* = 5/genotype – significance determined by unpaired Student’s t-test (t_8_ = 2.711, *p = 0.0266). Levels of βCaMKII binding to γCaMKII were normalized to respective inputs (1%). (**P**) Western blotting for γCaMKIIT287ph levels (normalized to total γCaMKII levels and Gapdh as loading controls) in dHPC from wildtype *vs*. γCaMKIIQ285A mutant mice. *n* = 6/genotype – significance determined by unpaired Student’s t-test (t_10_ = 2.240, *p = 0.0490). (**Q**) Western blotting for CREBS133ph levels (normalized to total CREB levels and Gapdh as loading controls) in dHPC from wildtype *vs*. γCaMKIIQ285A mutant mice. *n* = 6/genotype – significance determined by unpaired Student’s t-test (t_10_ = 2.361, *p = 0.0399). All bar plots presented as mean ± SEM. See Figure S8 for uncropped blots.

Given the relative immaturity of neuronal primary culture systems, as well as the relative absence of other relevant neural cell-types (e.g., glia), we next sought to characterize the *in vivo* protein dopaminylome in mouse brain. Adult tissues from mouse ventral tegmental area (VTA), nucleus accumbens (NAc), medial prefrontal cortex (mPFC) and dorsal hippocampus (dHPC) were chosen as brain regions of interest owing to their known DA-dependent physiology and their relevance to DA-mediated behaviors (33). Following Bio-CO labeling of mouse brain extracts, Streptavidin enrichment and LC-MS/MS, we identified a total of 1,557 uniquely dopaminylated substrates across these four brain regions, with VTA – which is enriched for dopaminergic neurons – displaying the largest number of dopaminylated proteins (1,281; Fig. 1A, Data S3), followed by NAc (737; Fig. 1B, Data S4), mPFC (719; Fig. 1C, Data S5) and dHPC (357; Fig. 1D, Data S6), the latter three of which receive dense (NAc, mPFC) or restricted (dHPC) (34) DA projections from VTA (as well as substantia nigra, which was not investigated here). Similar to our *in cellulo* results using primary cultured neurons, a large percentage of these substrates were found to be synaptic (both pre- and post-synaptic; SynGO 2024), involved in synaptic regulation (GO BP 2023) and were enriched in biological pathways known to be disrupted in brain disorders (DisGeNET) that are associated with altered dopamine signaling – e.g., schizophrenia, intellectual disability, Parkinson’s disease, etc. (Fig. 1E). While some of the dopaminylated proteins were found to be uniquely modified in a brain region-specific manner, many of the proteins were found to be commonly modified across brain structures, with 91 putatively modified substrates observed in all four brain regions (Fig. 1F). Among these 91 proteins were numerous important synaptic constituents/regulators, including Dlg4 (PSD-95), NMDA receptor subunits Grin1 and Grin2b, and three members of the Ca^2+^/calmodulin-dependent protein kinase family – αCaMKII (Camk2a), βCaMKII (Camk2b) and γCaMKII (Camk2g) (∂CaMKII, while expressed in brain, was only shown to be a substrate of dopaminylation in VTA), the latter three of which were validated in mouse brain extracts (dHPC) via Bio-CO labeling, enrichment and western blotting (Fig. 1G). To further confirm that our Bio-CO probe labeled endogenously dopaminylated proteins and not those that may be modified *in situ* following tissue/cell lysis (during which time TGM2 and DA may theoretically come into contact with proteins with which they may otherwise not interact *in vivo*), we performed a spike-in control experiment using recombinant, non-dopaminylated FLAG-tagged γCaMKII, which was added to tissue extracts post-lysis, followed by Bio-CO labeling, enrichment and western blotting for FLAG or γCaMKII. These results confirmed that the endogenous γCaMKII dopaminylation observed in Fig. 1A–D, G was not due to TGM2-mediated dopaminylation *in situ* (Fig. S2M). Next, to ensure that the dopaminylation observed for αCaMKII, βCaMKII and γCaMKII was direct (i.e., the proteins were not IP’d as a result of being bound to other dopaminylated proteins in the extracts), we performed *in vitro* TGM2 enzymatic assays on recombinant αCaMKII, βCaMKII and γCaMKII using monodansylcadaverine (MDC; an autofluorescent monoamine analog) with or without donor competition using excess DA. These results indicated that all three CaMKII proteins were direct substrates of TGM-dependent dopaminylation (Fig. 1H).

Given that our proteomics data robustly implicated roles for brain dopaminylation in synaptic development, regulation and plasticity, along with their relative enrichment in disease ontologies related to cognition (e.g., intellectual disability, autism spectrum disorder, etc.), we next wished to further dissect potential mechanistic roles for these dopaminylation events *in vivo*. One particular validated candidate of note, γCaMKII, had recently been shown to be member of a cluster of genes (*CAMK2G*) associated with memory in humans and was shown to at least partially explain variation in human hippocampal activity via fMRI (35). Furthermore, alternative splicing events in *CAMK2G* have been shown to be associated with autism (36), and γCaMKIIR292P mutations are known to result in profound synaptic deficits and intellectual disability in humans, with expression of γCaMKIIR292P in mice similarly yielding memory deficits (37, 38). Finally, in exhaustive work from the Tsien lab, γCaMKII was previously demonstrated to be a critical synaptic-to-nuclear signaling molecule, where it translocates from the cytosol to the cell membrane upon Ca^2+^-dependent activation of βCaMKII, resulting in its further shuttling with calmodulin (CaM) to the nucleus to phosphorylate the transcription factor CREB. These events, in turn, induce transcription at CREB target genes (39). Importantly, Ma *et al.,* demonstrated that γCaMKII shuttling to the cell membrane, which allows for direct interactions with βCaMKII, functions as a critical ‘checkpoint’ for signaling to proceed properly (39). Despite this incredibly thorough work, however, the signal for recruitment of γCaMKII to the synaptic membrane upon cellular activation was not identified. Given that γCaMKII can exist in the cytoplasm, the nucleus and at the synapse, we next aimed to examine in which sub-cellular compartment γCaMKII is dopaminylated – focusing now on the hippocampus given γCaMKII’s important regulatory roles in this region – to assess whether this modification may influence γCaMKII’s cellular signaling, events that are critical for hippocampal-dependent learning and memory. As such, we biochemically fractionated dHPC tissues from wildtype adult mice into nuclear-, cytosolic- and synaptosomal-enriched fractions, followed by Bio-CO labeling, enrichment and western blotting for γCaMKII. Our results indicated that γCaMKII dopaminylation was almost exclusively synaptic (Fig. 1I). We next explored which amino acid residue(s) within γCaMKII are direct substrates for monoaminylation. We again performed TGM2 enzymatic assays against recombinant human γCaMKII using MDC as a monoamine donor, followed by LC-MS/MS. While γCaMKII contains numerous glutamines (Q) (the primary amino acid substrate for transglutamination), we found that only one residue – γCaMKIIQ285 – was able to be modified *in vitro* (Fig. 1J). To confirm whether the specificity for Q285 monoaminylation was consistent across other CaMKII proteins, we similarly performed TGM2 enzymatic assays against αCaMKII, where we found that this same glutamine (Q284 in αCaMKII) was the primary site of monoaminylation (Fig. S3A–B). Since γCaMKIIQ285 exists within a conserved region of the CaMKII kinase family that functions as a Ca^2+^-CaM binding domain (Fig. 1K), we reasoned that dopaminylation of this glutamine may impact aspects of its cellular signaling.

## Synaptic γCaMKIIQ285dop contributes to γCaMKII membrane localization and downstream synaptic-to-nuclear signaling

As alluded to previously, γCaMKII is a synaptic-to-nuclear signaling protein that sequesters CaM in a threonine 287 phosphorylation (T287ph)-dependent manner, thereby promoting nuclear shuttling, CREB phosphorylation at serine 133 (ser133ph) and CREB-mediated transcription. Given that γCaMKIIQ285 is only two amino acid residues away from the autoinhibitory T287ph site, we hypothesized that γCaMKIIQ285dop may promote crosstalk interactions between these two PTMs to regulate γCaMKII signaling through this pathway. To further investigate this potential crosstalk mechanism *in vivo*, we next employed CRISPR-based gene editing in mice to generate whole-body γCaMKIIQ285A – a mutation that does not impact γCaMKII’s intrinsic kinase activity *in* vitro; (Fig. S3C) – knock-in mice (Fig. S3D–G), which completely abolished endogenous γCaMKIIQ285dop levels in mutant animals (Fig. 1L). Next, to assess what impact eliminating γCaMKIIQ285dop may have on γCaMKII signaling in brain, we performed a series of biochemical assessments on dHPC tissues from wildtype *vs*. γCaMKIIQ285A mice, which demonstrated that while the mutation does not impact total γCaMKII levels (Fig. 1M; nor total levels of protein dopaminylation in dHPC – Fig. S3H), loss of the mark resulted in reduced trafficking of γCaMKII to the synapse (Fig. 1N), decreased binding of γCaMKII to βCaMKII (Fig. 1O), attenuation of γCaMKIIT287ph (Fig. 1P) and reduced levels CREBS133ph (Fig. 1Q). To further validate the potential impact of reducing γCaMKIIQ285dop levels on CREB-mediated transcription, we transduced primary hippocampal neurons isolated from wildtype *vs*. γCaMKIIQ285A mice with a lentivirus expressing a CREB response element (CRE) driving a luciferase reporter gene, and then measured CREB-mediated transcription directly following treatment with 500 nM DA (*vs*. vehicle). While DA treatments in wildtype neurons resulted in a predictable increase in CREB-induced transcription, we observed a significant attenuation of this activity in γCaMKIIQ285A neurons (Fig. S3I). These results indicated that γCaMKIIQ285dop contributes importantly to γCaMKII’s synaptic-to-nuclear signaling and downstream CREB-mediated transcription, phenomena that may further impact neuronal gene expression, physiology and/or behavior.

## γCaMKIIQ285dop influences neuronal gene expression in hippocampus and contributes to CA1 neuron excitability

**Fig. 2:**
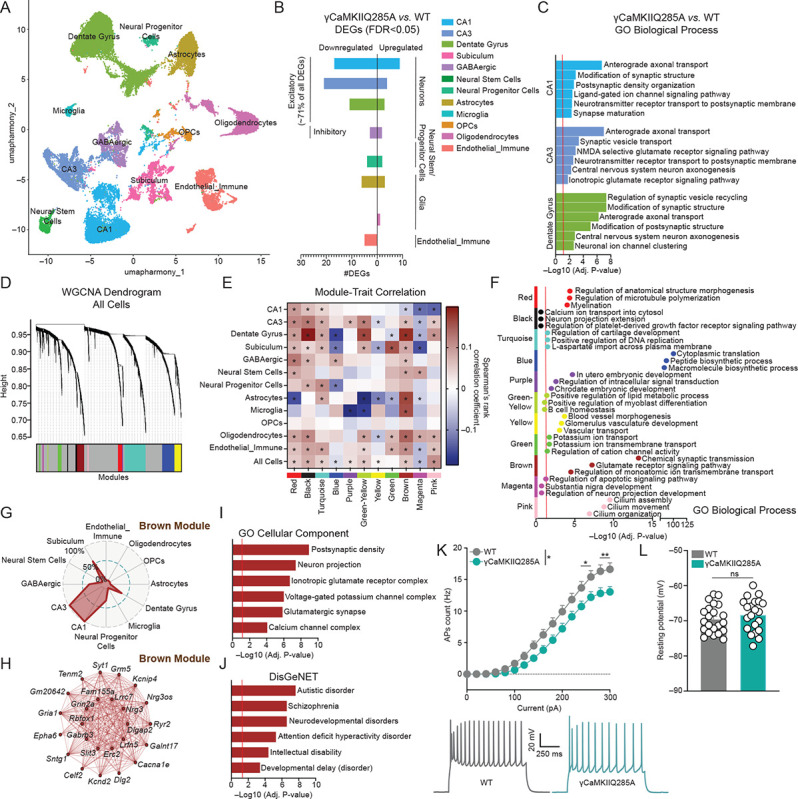
Synaptic γCaMKIIQ285dop is required for normal hippocampal gene expression and intrinsic CA1 pyramidal neuron excitability. (**A**) Uniform manifold approximation and projection (UMAP), colored by major cell-type (*n* = 3 wildtype; *n* = 4 γCaMKIIQ285A). (**B**) Number of DEGs observed by cell-type from pseudobulk analysis (FDR<0.05). (**C**) Gene set enrichment analysis (GO Biological Process) for DEGs identified in CA1, CA3 and Dentate Gyrus excitatory neurons from pseudobulk analysis (FDR<0.05; Benjamini-Hochberg). (**D**) Dendrogram showing co-expression modules identified by weighted gene correlation network analysis (WGCNA). (**E**) Heatmap of co-expression module correlation with mutation (γCaMKIIQ285A) trait by cell-type. * indicates Adj. P <. 05 significance of correlation. (**F**) enrichR-based gene ontology (GO Biological Process) analysis for genes in significant co-expression modules (FDR<0.05; Benjamini-Hochberg). (**G**) Radar plots of WGCNA-identified Brown module expression by annotated cell cluster. % expression of module genes by cell-type is noted. (**H**) Network underlying the top 25 hub genes in the Brown module. Each node represents a hub gene, and each edge represents the co-expression relationship between two genes in the network. The top 10 hub genes are in the center of the plot, while the remaining 15 are in the outer circle. (**I**) enrichR-based gene ontology (GO Cellular Component) analysis for genes in brown module (FDR<0.05; Benjamini-Hochberg). (**J**) enrichR-based gene ontology (DisGeNET) analysis for genes in brown module (FDR<0.05; Benjamini-Hochberg). (**K**) The number of evoked action potentials (APs) in dHPC CA1 pyramidal neurons of wildtype (*n* = 22 neurons from 5 mice) *vs*. γCaMKIIQ285A (*n* = 21 neurons from 4 mice) in response to increasing depolarizing current steps. Representative membrane responses are provided from dHPC CA1 pyramidal neurons in response to a 280 pA current injection in wildtype or γCaMKIIQ285A mutant mice. Significance was determined via two-way ANOVA (main effect of interaction between current injection and genotype; F_15, 656_ = 1.814) with subsequent post hoc analysis (Sidak’s MC test: *p = 0.0425 or 0.0182, **p = 0.0064 or 0.0047). (**L**) Resting membrane potential of dHPC CA1 pyramidal neurons of wildtype (*n* = 22 neurons from 5 mice) *vs*. γCaMKIIQ285A (*n* = 21 neurons from 4 mice). Significance was determined by unpaired Student’s t-test (t_41_ = 0.9639, *p* = 0.3407). All bar/line plots presented as mean ± SEM.

We next wished to examine how reducing levels of the mark affects gene expression across hippocampal cell-types. For this, we performed single-nuclei RNA-sequencing (snRNA-seq) on adult dHPC tissues from wildtype *vs*. γCaMKIIQ285A mutant mice. After removing doublets and filtering out low quality cells (see Fig. S4A–D for relevant quality control metrics post-filtering), we obtained a total of 33,983 nuclei for downstream processing (wildtype = 16,256; γCaMKIIQ285A = 17,727). Using both manual curation and label transfer with high-resolution validated annotations from previously published hippocampus datasets from the Allen Brain Atlas (40), we annotated 12 major cell-type groups, including excitatory neuron subtypes (CA1, CA3, Dentate Gyrus and Subiculum; 20,510 nuclei, 60.35% of total), GABAergic subtypes (2,040 nuclei, 6.00% of total), neural stem cells and neural progenitor cells (1,562 nuclei, 4.59% of total), glial cell-types (astrocytes, microglia, oligodendrocyte progenitor cells/OPCs and oligodendrocytes; 7,384 nuclei, 21.72% of total) and endothelial/immune cells (2,487 nuclei, 7.31% of total) (Fig. 2A, Fig. S5A–C). We next used pseudobulk aggregation and differential expression testing to identify differentially expressed genes (DEGs; FDR<0.05) between wildtype *vs*. γCaMKIIQ285A mutant mice within each annotated cell-type. Consistent with γCaMKII being broadly expressed across all cell-types (Fig. S6A), this analysis showed that while many cell-clusters displayed at least modest levels of differential gene expression as a result of reducing γCaMKIIQ285dop levels, excitatory neuron clusters (specifically CA1, CA3 and Dentate Gyrus) exhibited the most robust alterations (~71% of all DEGs across all cell-types), with the vast majority of these genes being downregulated in mutant animals (Fig. 2B, Data S7); note that *Camk2g* expression itself was not found to be affected by the mutation, which is consistent with our western blotting results in Fig. 1. Gene set enrichment analysis with GO Biological Process datasets further demonstrated that gene sets related to synaptic function (e.g., anterograde axonal transport, synaptic vesicle transport, ionotropic glutamate receptor signaling pathway, ligand-gated ion channel signaling pathway, etc.) were significantly differentially enriched in CA1, CA3 and Dentate Gyrus between the two conditions (Fig. 2C).

Given the potential complexity and interconnectedness of transcriptional changes that may occur as a result of reducing γCaMKII dopaminylation, we next sought to examine the effects of the γCaMKIIQ285A mutation on network-level gene expression, rather than individual gene features. Therefore, we employed weighted gene correlation network analysis on our snRNA-seq dataset (hdWGCNA package; version 0.3.03) to identify cell-type specific modules of highly co-expressed genes across both conditions (Fig. 2D). We next evaluated if these identified modules were relevant to the γCaMKIIQ285A mutation by calculating the correlation between the expression module genes and the trait of mutation across identified cell-types (Fig. 2E). We found that several modules were significantly correlated with γCaMKIIQ285A, and that the directionality of module gene expression was cell-type specific. For example, the Brown module was positively correlated with the mutation in all cell-types (including CA3 and Dentate Gyrus) except for CA1 cells, where genes in this module showed a negative correlation with the mutation (corresponding to reduced expression in the γCaMKIIQ285A group). To better understand the functional relevance of these significantly correlated modules in excitatory neuron sub-types, we next performed gene ontology enrichment analysis (Fig. 2F). Of particular interest, the Brown module was enriched for genes related to synaptic function (e.g., chemical synaptic transmission, glutamate receptor signaling pathway, regulation of monoatomic ion transmembrane transport, etc.). Furthermore, genes in the Brown module were mainly expressed in excitatory neurons (CA1 and CA3 – Fig. 2G–H; see Fig. S6B for module enrichment across other annotated cell-types, and Data S8 for lists of genes enriched in each module). These data suggested that genes within the Brown module may be uniquely disrupted in CA1 excitatory neurons by loss of γCaMKIIQ285dop and may, in turn, contribute to synaptic dysregulation in these neurons. To examine this possibility further and to pinpoint specific cellular components and disease ontologies related to these disrupted genes in CA1, we performed an expanded GO analysis on genes in the Brown module focusing on cellular localization/components (GO Cellular Component) and disease ontologies (DisGeNET). These analyses revealed significant correlations with synaptic structures, such as ionotropic glutamate receptor complexes, voltage-gated K^+^ channel complexes and Ca^2+^-channel complexes (Fig. 2I), as well as disease ontologies related to cognitive impairment (e.g., intellectual disability, autistic disorder, developmental delay; Fig. 2J).

In total, these sequencing data indicated significant deficits in dHPC excitatory neuron gene expression in mutant mice, as well as unique patterns of transcriptional dysregulation in CA1 that may be predicted to result in abnormal synaptic architecture (e.g., postsynaptic density) and/or disruptions in voltage-gated potassium/ionotropic glutamate receptor signaling. Given this, as well as previous data implicating γCaMKII in human hippocampal activity^33^, we next wished to explore what impact disruptions of the mark may have on intrinsic electrophysiological properties of CA1 neurons. For this, we performed *ex vivo* patch-clamp recordings on CA1 pyramidal neurons in wildtype *vs*. γCaMKIIQ285A mice to assess the impact of γCaMKIIQ285dop loss on intrinsic neuronal excitability. As predicted, these results indicated that dHPC CA1 intrinsic neuronal excitability was significantly attenuated in mutant animals (Fig. 2K; note that the resting membrane potential of CA1 neurons was not impacted by the mutation – Fig. 2L). Taken together, these data demonstrated that the transcriptional deficits induced by loss of γCaMKIIQ285-dopmediated synaptic-to-nuclear signaling directly impacted normal cellular physiology, which, in turn, may also be predicted to result in deficits of other dHPC-dependent phenotypes (i.e., behavior).

## γCaMKIIQ285dop in dHPC is critical for contextual memory formation

**Fig. 3:**
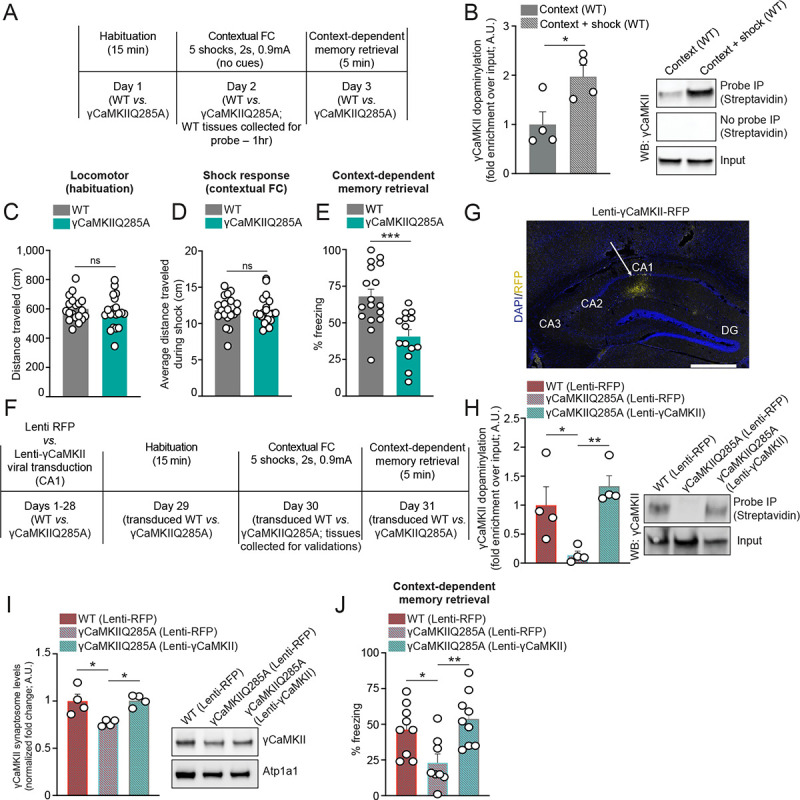
γCaMKIIQ285dop is induced during learning and contributes to contextual memory. (**A**) Timeline of contextual fear conditioning experiments in wildtype *vs*. γCaMKIIQ285A mutant mice. (**B**) Bio-CO probe-mediated bioorthogonal labeling of dopaminylated γCaMKII from dHPC of wildtype mice + (context + shock) *vs*. − (context alone) contextual fear conditioning training (1 hr post-training). Streptavidin IPs were performed for − *vs*. + probe conditions and γCaMKII levels were normalized to respective inputs (1%). *n* = 4/genotype – significance determined by unpaired Student’s t-test (t_6_ = 2.845, *p = 0.0294). (**C**) Locomotor activity during context habituation prefear conditioning for wildtype (*n* = 19) *vs*. γCaMKIIQ285A mutant (*n* = 20) mice. Significance determined by unpaired Student’s t-test (t_37_ = 0.6553, *p* = 0.5163). (**D**) Average locomotor activity across shock pairing trials for wildtype (*n* = 19) *vs*. γCaMKIIQ285A mutant (*n* = 20) mice. Significance determined by unpaired Student’s t-test (t_37_ = 0.1853, *p* = 0.8540). (**E**) Context-dependent memory retrieval 24 hr post-training for wildtype (*n* = 17) *vs*. γCaMKIIQ285A mutant (*n* = 14) mice. Significance determined by unpaired Student’s t-test (t_29_ = 4.002, ***p = 0.0004). (**F**) Timeline of contextual fear conditioning experiments in wildtype *vs*. γCaMKIIQ285A mutant mice following intra-dHPC transduction with either Lenti-RFP or Lenti-γCaMKII. (**G**) IHC/IF image of Lenti-γCaMKII-RFP targeting to dHPC (CA1). DAPI was used as a counterstain to mark nuclei. Scale bar is set to 500 μm. (**H**) Bio-CO probe-mediated bioorthogonal labeling of dopaminylated γCaMKII from dHPC of wildtype *vs*. γCaMKIIQ285A mutant mice transduced with either Lenti-RFP or Lenti-γCaMKII. γCaMKII levels were normalized to respective inputs (1%). *n* = 4/genotype – significance determined by one-way ANOVA (F_2,9_ = 8.207, **p = 0.0094), followed by post hoc analysis (Tukey’s MC test; *p = 0.0468, **p = 0.0088). (**I**) Western blotting for total γCaMKII levels in dHPC synaptic fractions (normalized to Atp1a1 as a loading control) from wildtype *vs*. γCaMKIIQ285A mutant mice transduced with either Lenti-RFP or Lentig-γCaMKII. *n* = 4/genotype – significance determined by one-way ANOVA (F_2,9_ = 8.449, **p = 0.0086), followed by post hoc analysis (Tukey’s MC test; *p = 0.0163 for wildtype RFP *vs*. Q285A RFP, *p = 0.0143 for Q285A RFP *vs*. Q285A γCaMKII). (**J**) Context-dependent memory retrieval 24 hr post-training for wildtype (*n* = 9; RFP) *vs*. γCaMKIIQ285A mutant mice transduced with either Lenti-RFP (*n* = 8) or Lenti-γCaMKII (*n* = 9). Significance determined by one-way ANOVA (F_2,23_ = 6.676, **p = 0.0052), followed by post hoc analysis (Tukey’s MC test; *p = 0.0350, **p = 0.0049). All bar plots presented as mean ± SEM. See Figure S8 for uncropped blots.

Given roles for γCaMKIIQ285dop in mediating γCaMKII trafficking to the synapse (and regulation of downstream signaling), hippocampal transcription related to synaptic function and intellect, and normal neuronal physiology, we next sought to explore what impact disrupting γCaMKIIQ285dop may have on cognition. We first asked whether levels of γCaMKIIQ285dop may be altered in response to a learned experience by putting wildtype mice through contextual fear conditioning. In this paradigm, animals were first habituated to a neutral environment before receiving a series of five intermittent shocks at random intervals (two seconds each, 0.9 mA) over the course of the training session (Fig. 3A). One hour following the final shock, dHPC tissues from trained (context + shock) *vs*. untrained (context only) animals were collected and subjected to Bio-CO labeling, enrichment and western blotting for γCaMKII (Fig. 3B) or βCaMKII (Fig. S7A). These results demonstrated that γCaMKIIQ285dop, but not βCaMKII dopaminylation, levels were induced in dHPC by context + shock training, suggesting that learning-induced increases in γCaMKIIQ285dop may contribute to cellular signaling/transcription that might be necessary for contextual learning and/or formation of contextual memories. To explore this further, wildtype *vs*. γCaMKIIQ285A mice were put through the same contextual fear conditioning paradigm and behavioral responses (locomotor behavior during habituation, shock response, learning acquisition and context-dependent memory retrieval) were assessed. Consistent with our molecular and biochemical data, we found that while γCaMKIIQ285A animals did not display alterations in locomotor behavior during habituation (Fig. 3C) or directly following shocks (Fig. 3D), they did display deficits in both learning acquisition [Fig. S7B; note that mutant mice did learn (reaching a threshold of ~60% freezing by trial five), yet at a slower rate] and context-dependent memory retrieval (Fig. 3E).

Although these data clearly indicated that γCaMKIIQ285dop plays a critical role in contextual memory formation, it was important to consider that the γCaMKIIQ285A knock-in mice carry this mutation throughout their entire body, making it difficult to ascertain whether such memory deficits are indeed precipitated directly through alterations in hippocampal function. As such, we next wished to examine whether rescue of γCaMKIIQ285dop specifically within dHPC (focusing on CA1, a brain region/neuronal cell cluster most robustly impact by loss of the mark – see Fig. 2) could restore normal contextual memory in mutant mice. To do so, we transduced γCaMKIIQ285A mutant mice with a lentivirus expressing wildtype γCaMKII (co-expressing the fluorescent reporter RFP; Lenti-γCaMKII) *vs*. a lentivirus expressing RFP alone (Lenti-RFP), followed by 28 days of incubation to allow for maximal viral expression and then exposure to contextual fear conditioning. A separate group of wildtype mice were transduced with Lenti-RFP to serve as an additional control in our experimental design (Fig. 3F). In an independent cohort of animals not exposed to contextual fear conditioning, we confirmed appropriate targeting of our viral vectors to CA1 (Fig. 3G) and demonstrated that add-back of wildtype γCaMKII (*vs*. RFP) fully restores γCaMKIIQ285dop levels in dHPC to that of wildtype levels (Fig. 3H). We additionally explored whether such rescue may also be sufficient to restore deficits in γCaMKII trafficking to the synapse, which was previously observed in γCaMKIIQ285A mice. Our results indicated that this rescue approach was sufficient to restore γCaMKII synaptic trafficking to that of wildtype levels (Fig. 3I). Finally, we assessed whether restoring γCaMKIIQ285dop levels in CA1 may be sufficient to rescue contextual memory deficits observed in mutant animals. Following context re-exposure 24 hr post-training, mutant mice transduced with Lenti-γCaMKII were observed to display freezing levels similar to that of wildtype mice (transduced with Lenti-RFP), significantly reversing the memory deficits observed in γCaMKIIQ285A expressing Lenti-RFP alone (Fig. 3J).

## DISCUSSION

We feel that the data presented here provide a compelling case for protein dopaminylation as an important, yet previously undescribed, synaptic signaling mechanism in brain, with DA not only acting through cell surface-bound receptors but also infiltrating post-synaptic cells to directly modify proteins therein. We found that attenuation of γCaMKIIQ285dop – which is near exclusively synaptic – in dHPC results in disruptions in γCaMKII’s synaptic-to-nuclear signaling and its influence on neural transcriptional patterning, neuronal excitability, and context-dependent learning and memory. Based upon these data, we now hypothesize that upon dopamine uptake into cells and Ca^2+^-dependent cellular activation, TGM2 dopaminylates γCaMKII at position Q285 to promote γCaMKII trafficking to the membrane where it can then engage with βCaMKII. Reaching this previously described cellular ‘checkpoint’ is then critical for the proper downstream signaling of γCaMKII. Importantly, restoration of γCaMKIIQ285dop specifically within CA1 of mutant mice lacking the PTM rescues the expression of contextual fear memory in whole-body knock-in mice. We believe that this regional specificity helps to bolster the argument that γCaMKIIQ285dop functions as an acute mediator of learning and memory formation, and lends evidence to support the assumption that these effects are indeed dopamine-dependent, yet in a non-canonical, DA receptor-independent manner. Given that VTA-to-dHPC-dependent DA signaling has previously been shown to contribute importantly to contextual learning (34), it stands to reason that some of these effects may be mediated by dopaminylation of γCaMKII in dHPC. While compelling in and of itself, we wish to emphasize that similar dopaminylation-based regulation of the synaptic proteome is likely generalizable to many protein substrates beyond that of just γCaMKII, with these additional dopaminylated substrates (for which modified sites may exist both intra- and extracellularly) likely also contributing to important aspects of protein signaling and/or downstream gene expression, physiology, and behavior. For example, the dopamine transporter (DAT; Slc6a3) – a membrane-spanning protein that is critical for DA reuptake from the synapse to the cytosol of dopaminergic neurons – was identified as a substrate of dopaminylation in NAc (likely modified at VTA-to-NAc projection terminals). While the specific resides that are modified within DAT have yet to be elucidated, the possibility that dopaminylation of this transporter may contribute to its reuptake mechanism and, in turn, cellular physiology and behavior, is certainly intriguing. Another example is that of TGM2, which was found to be a substrate of dopaminylation in VTA, NAc and dHPC. Although much remains to be learned regarding how TGM2’s enzymatic activity and/or sub-cellular distribution are controlled within cells, such ‘automonoaminylation’ is perhaps suggestive of a potential monoamine sensing mechanism that may contribute to its regulation of monoaminylation states during periods of dynamic monoamine fluctuations in brain (and beyond). Finally, while our current data indicate a critical role for synaptic dopaminylation in adaptive brain plasticity (e.g., learning and memory), they may also implicate these phenomena in pathologies associated with altered monoaminergic signaling, although future studies will be necessary to fully elucidate these possibilities.

Importantly, this work does present with a few challenges/outstanding questions that will need to be addressed in follow up studies. First, while our *in vitro* and *in cellulo* validations of Bio-CO specificity towards dopaminylated *vs*. noradrenylated substrates indicated that the probe does not efficiently label noradrenylated proteins under the reaction conditions used in this study, it remains possible that noradrenylated substrates in brain (which have yet to be characterized owing to a lack of available tools/reagents for their investigation) are more robustly modified by TGM2 *vs*. that observed following NE treatments in primary neuronal culture. As such, future investigations will be needed to develop new tools that can fully distinguish between endogenously dopaminylated *vs*. noradrenylated protein *in vivo* Second, we acknowledge that our mutational approach perhaps represents an imperfect solution for testing the necessity of γCaMKIIQ285dop under normal physiological conditions, as the Q285A mutation might be expected to disrupt all possible monoaminylation events at this site (i.e., possibly disrupting serotonylation, histaminylation, etc.) if they are to occur *in vivo*. Unfortunately, however, the field currently lacks the tools necessary to *de novo* generate γCaMKIIQ285dop in brain/cells for causal analyses, although future studies implementing intein-based chemical methodologies (41), for example, may hold promise in this regard. In addition, while we found that the γCaMKIIQ285A mutation does not impact γCaMKII expression (RNA or protein) in mice or its intrinsic kinase activity *in vitro*, it remains possible that the mutation may affect other aspects of γCaMKII function *in vivo* that are not fully captured in our analyses and could be independent of its ability to be dopaminylated. Future studies may be needed to account for these additional possibilities. Finally, classical pathways for DA uptake via canonical transporter mechanisms (e.g., via SLC6A3/DAT) are believed to be restricted to certain populations of glial cells and the terminals of dopaminergic neurons, leaving little room for their utility in post-synaptic DA uptake, as suggested here (42). However, it is important to note that a myriad of non-canonical, high-capacity/low affinity monoamine transporters (e.g., organic cation transporters 2 and 3/OCT2 and OCT3, plasma monoamine transporter/PMAT) have been shown to be broadly expressed in brain and may also contribute to DA uptake into non-dopaminergic cells (43, 44). Specifically relevant to protein monoaminylation, it was recently shown that OCT3 – which is enriched at both plasma and nuclear membranes in neural cells (45) – plays a direct and causal role in the transport of 5-HT into astrocytes to promote the enzymatic addition of serotonylation on histone H3 (46). These previous findings suggest OCT3 as a prime target for future studies related to synaptic protein dopaminylation. Investigating these transporters, as well as potential alternative mechanisms of DA internalization (e.g., receptor internalization), in future studies will be paramount to dissecting the precise kinetics of dopaminylation events in non-monoaminergic cells in brain and may even represent novel targets for future therapeutic interventions aimed at treating pathologies associated with altered monoaminergic signaling.

Undoubtedly, additional monoamines – such as 5-HT, NE and/or histamine – will also be capable of similar actions in brain, including at the synapse, with such non-canonical monoaminylation events possibly representing a fundamental shift in our understanding of monoaminergic signaling writ-large. This is not to argue that canonical means of monoamine signaling/neuromodulation are not fundamentally important to cellular physiology, as they clearly are, but rather that protein monoaminylation likely also contributes importantly to cellular signaling and will require future intensive investigation. As such, with the creation of future tools aimed at targeting and dissecting mechanistic roles for these protein monoaminylation PTMs *in vivo*, we strongly believe that gaining a more detailed understanding of these phenomena may help to illuminate numerous mechanisms of brain physiology and disease that remain unresolved.

## Supplementary Material

Supplement 1

1

## Data Availability

The snRNA-seq data generated in this study have been deposited in the National Center for Biotechnology Information Gene Expression Omnibus (GEO) database under accession number GSE277319. Mass spectrometry proteomics data have been deposited to the ProteomeXchange Consortium via the PRIDE partner repository with the dataset identified as PXD055995. We declare that the data supporting findings for this study are available within the article and Supplementary Materials. Related data and code are available from the corresponding author upon reasonable request. No restrictions on data availability apply.
