# Supplementary material for: Hippocampal γCaMKII dopaminylation promotes synaptic-to-nuclear signaling and memory formation
Source: bioRxiv. 2024 Sep 24:2024.09.19.613951. Preprint. [Version 2] doi: 10.1101/2024.09.19.613951 (PMC11430047; doi:10.1101/2024.09.19.613951)
Supplement: 1 [file NIHPP2024.09.19.613951v2-supplement-1.pdf]

bioRxiv preprint doi: https://doi.org/10.1101/2024.09.19.613951; this version posted September 24, 2024. The copyright holder for this preprint (which was not certified by peer review) is the author/funder, who has granted bioRxiv a license to display the preprint in perpetuity. It is made available under aCC-BY 4.0 International license.

Supplementary Materials for

Hippocampal γCaMKII dopaminylation promotes synaptic-to-nuclear signaling and memory formation

Andrew F. Stewart *et al.*

Corresponding author: Ian Maze, ian.maze@mssm.edu

The file includes:

Materials and Methods
Figs. S1 to S8
Supplementary Data Descriptions
References

Other Supplementary Material for this manuscript includes the following:

Data S1-S8

bioRxiv preprint doi: https://doi.org/10.1101/2024.09.19.613951; this version posted September 24, 2024. The copyright holder for this preprint (which was not certified by peer review) is the author/funder, who has granted bioRxiv a license to display the preprint in perpetuity. It is made available under aCC-BY 4.0 International license.

MATERIALS AND METHODS

Synthesis of Bio-Co probe

Materials

^{1}H spectra were acquired on a Bruker DRX-600 spectrometer at 600 MHz for 1H. Thin layer chromatography (TLC) was performed on silica coated aluminum sheets (thickness 200 µm) or alumina coated (thickness 200 µm) aluminum sheets supplied by Sorbent Technologies, and column chromatography was carried out on Teledyne ISCO combiflash equipped with a variable wavelength detector and a fraction collector using a RediSep Rf high performance silica flash columns by Teledyne ISCO. LCMS/HPLC analysis for purity determination and HRMS was conducted on an Agilent Technologies G1969A high-resolution API-TOF mass spectrometer attached to an Agilent Technologies 1200 HPLC system. Samples were ionized by electrospray ionization (ESI) in positive mode. Chromatography was performed on a 2.1 × 150 mm Zorbax 300SBC18 5-µm column with water containing 0.1% formic acid as solvent A and acetonitrile containing 0.1% formic acid as solvent B at a flow rate of 0.4 mL/min. The gradient program was as follows: 1% B (0−1 min), 1−99% B (1−4 min), and 99% B (4−8 min). The temperature of the column was held at 50 °C for the entire analysis. The purity of all the compounds was ≥95%. N-[(1R,8S,9s)-Bicyclo[6.1.0]non-4-yn-9-ylmethyloxycarbonyl]-1,8-diamino-3,6-dioxaoctane was purchased from Sigma-Aldrich (CAS #: 1263166-93-3) and EZ-link desthiobiotin was obtained from ThermoFisher (CAS #: 80750-24-9).

Experiment

((1R,8S,9s)-bicyclo[6.1.0]non-4-yn-9-yl)methyl(2-(2-(2-(6-(5-methyl-2-oxoimida-zolidin-4-yl)hexanamido)ethoxy)ethoxy)ethyl)carbamate: A solution of ((1R,8S,9s)-bicyclo[6.1.0]non-4-yn-9-yl)methyl (2-(2-(2-aminoethoxy)ethoxy)ethyl)carbamate (133 mg, 1.23 eq, 410 µmol), 2,5-

bioRxiv preprint doi: https://doi.org/10.1101/2024.09.19.613951; this version posted September 24, 2024. The copyright holder for this preprint (which was not certified by peer review) is the author/funder, who has granted bioRxiv a license to display the preprint in perpetuity. It is made available under aCC-BY 4.0 International license.

dioxopyrrolidin-1-yl 6-(5-methyl-2-oxoimidazolidin-4-yl)hexanoate (103.7 mg, 1.0 eq, 333.1 μmol) and triethylamine (139 μL, 3.0 eq, 999.2 μmol) in a minimum amount of DMF (0.5 mL) was stirred at 25 °C for 3 hours. After 3 hours, the reaction mixture was extracted with ethyl acetate and water. The ethyl acetate layer was collected, dried with sodium sulfate and filtered to give a filtrate. The filtrate was mixed with silica gel and dried by rotovap vacuum. The dry solid mixture was purified (solid loading) by the normal phase (*Teledyne ISCO* 12 gram column cat# 69-2203-312; 100% DCM for 3 minutes to 5% MeOH in DCM for 10 minutes to 10% MeOH in DCM for 6 minutes) to afforded ((1R,8S,9s)-bicyclo[6.1.0]non-4-yn-9-yl)methyl(2-(2-(2-(6-(5-methyl-2-oxoimidazolidin-4-yl)hexanamido)ethoxy)ethoxy) ethyl)carbamate (yield: 65 mg; 37.5%). All fractions were monitored by TLC. After development, (10% MeOH in DCM), TLC plates were stained with potassium permanganate and heating. Some impurities and the desired product were stained and appeared as brown spots. These fractions containing compounds that stained on TLC were then defined by direct MS only (the desired product unable to be detected at wavelength 220 nm and 254 nm). TLC Rf: 0.5 (10% MeOH in DCM). ^{1}H NMR (MeOD-d_4, 600 MHz) δ 4.07-4.04 (m, 1H), 3.74-3.70 (m, 1H), 3.62-3.57 (m, 1H), 3.52 (broad s, 4H), 3.44 (t, J = 5.19 Hz, 4H), 3.26 (t, J = 5.42 Hz, 2H), 3.19 (m, 2H), 2.21-2.07 (m, 2H), 2.11 (t, J = 7.45 Hz, 2H), 1.94-1.90 (m, 1H), 1.82-1.71 (m, 1H), 1.57-1.56 (m, 2H), 1.55-1.51 (m, 3 H), 1.42-1.38 (m, 2H),1.36-1.32 (m, 1H), 1.30-1.24 (m, 3H),1.23-1.19 (m, 3H),1.00 (d, J = 6.46 Hz, 3H), 0.89-0.77 (m, 2H). LCMS (ESI^{+}): m/z 521.3634 $(M+H)^{+}$.

Animals

Wildtype adult male mice (7-week-old) and pregnant females (E15; used for primary neuron culture experiments) (C57BL/6J) were purchased from The Jackson Laboratory. Homozygous γCaMKIIQ285A mice (bred to homozygosity) *vs.* wildtype littermate controls were generated as

bioRxiv preprint doi: https://doi.org/10.1101/2024.09.19.613951; this version posted September 24, 2024. The copyright holder for this preprint (which was not certified by peer review) is the author/funder, who has granted bioRxiv a license to display the preprint in perpetuity. It is made available under aCC-BY 4.0 International license.

described below. Adult mice were group housed (5 per cage) and pregnant females singly-housed on a 12-hour light/dark cycle (lights on from 7:00 A.M. to 7:00 P.M.) at constant temperature (23 ºC) with *ad libitum* access to food and water. Adult male mice (7-10 weeks old) were used for all experiments outlined in this manuscript and were sacrificed by rapid decapitation, with the exception of those involving primary cultured neurons (see below). All animal protocols were approved by the IACUC at the Icahn School of Medicine at Mount Sinai (ISMMS).

Generation of γCaMKIIQ285A knock-in mutant mice

The γCaMKIIQ285A knock-in mice were generated at the Stem Cell Engineering Core and the Mouse Genetics Core at the Icahn School of Medicine at Mount Sinai using CRISPR/Cas editing, as described in **Figure S3D**. Briefly, a synthetic single guide RNA (sgRNA) (Synthego) and recombinant *S. pyogenes* Cas9 nuclease (IDT, 1081059) were preassembled as a ribonucleoprotein (RNP) and injected into C57BL/6J blastocysts, together with a 200-mer single-stranded oligodeoxynucleotide (ssODN) carrying the CA>GC to generate the Q285A mutation and an ectopic HhaI recognition site used for screening. Founder mice were screened by restriction fragment length polymorphism (RFLP) using primers external to the ssODN (F1 and R1) and HhaI digestion of the PCR product to identify mice that underwent homology directed repair (HDR) after CRISPR/Cas9-induced double strand break (**Figure S3E**). Mice 7, 12, 41, 24, 31 and 51 showed an RFLP pattern compatible with an HDR event. To further validate the successful HDR event, mice 12, 31, 41 and 51 were subjected to targeted next generation sequencing (NGS) using primers F2 and R2. The sequencing data were analyzed with CRISPResso tool (*47*), and the results revealed that mice 12 and 41 carried the expected CA>GC change, but also an unwanted GA deletion as result of a nonhomologous end-joining (NHEJ) (**Figure S3F**). To determine if the GA deletion was on the same allele as the CA>GC change, the F2/R2 amplicon was cloned into a

bioRxiv preprint doi: https://doi.org/10.1101/2024.09.19.613951; this version posted September 24, 2024. The copyright holder for this preprint (which was not certified by peer review) is the author/funder, who has granted bioRxiv a license to display the preprint in perpetuity. It is made available under aCC-BY 4.0 International license.

sequencing vector (pCR Blunt II-TOPO), and the plasmids derived from single *E. coli* clones were subjected to Sanger sequencing. The results demonstrated that mouse 41 carried the CA>GC change and the GA deletion on different alleles. Mouse 41 was therefore used as the founder for the γCaMKIIQ285A mouse colony (**Figure S3G**). To maintain the colony and identify mice carrying the γCaMKIIQ285A allele, mice were screened using the Q285A_F and R2 primers (specific for the HDR allele), as provided in the Table below.

Mouse genotyping strategy

DNA for genotyping was isolated from tail/digit biopsies. Tissues biopsies were digested in 200 µl of DirectPCR Lysis Reagent (Tail) (Viagen Biotech Inc, CA, USA) and 0.2 mg of proteinase K for 12–15 h at 55 °C followed by 1 hour at 85 °C for proteinase K inactivation. The digested tissues were directly used for PCRs. Thirty-five cycles of PCR amplification were carried out with a hybridization temperature of 60 °C.

sgRNA	GTAAGCACTCTACCGTCTCT
ssODN 200-mer donor	GATGGGGGAAACAGAGGCAGGGGATCTGTGATGGAA<br>ATCTCACAGGCCTTTCTGTTTGACTTCCAGCAACGGT<br>CTACGGTGGCATCCATGATGCATCGCGCAGAGACGG<br>TAGAGTGCTTACGCAAATTCAACGCCCGGAGAAAAC<br>TGAAGGTAGGCACTAGCCTTCTGCATCGCTGGCCTCC<br>TTGTAGCTCCTGCCTGGT
F1 primer	GAAGCAGTCGCTGCATCAGG
R1 primer	GGCCCACTCACCTCTGTAGAG
F2 primer	CGTGGCCTCGTCTGGACAGTACTG

bioRxiv preprint doi: https://doi.org/10.1101/2024.09.19.613951; this version posted September 24, 2024. The copyright holder for this preprint (which was not certified by peer review) is the author/funder, who has granted bioRxiv a license to display the preprint in perpetuity. It is made available under aCC-BY 4.0 International license.

R2 primer	CGGGAGAGAGAGTCCCGTGAG
Q285A_F primer	GCATCCATGATGCATCGCGC

Primary mouse neuronal culture

E18 pregnant dams were anesthetized with isofluorane and then euthanized, followed by collection of embryos. Embryonic brains were extracted and microdessicted to isolate corticostriatal or hippocampal neurons. Cells were incubated in trypsin at 37 ºC for 10 minutes, followed by trituration into a single-cell suspensions. Cells were then plated onto Poly-D-Lysine coated plates in DMEM+Glutamax (Gibco, 10566016), supplemented with penicillin/streptomycin (Gibco, 15140122) and 10% fetal bovine serum (Gibco, 26140079) at appropriate densities for given assays. Following 24 hours, media was aspirated and replaced with Neurobasal Plus Neuronal Culture System (Gibco, A3653401), supplemented with CultureOne (Gibco, A3320201) to suppress glial growth. Relevant experiments were performed on DIV14.

Protein purification

Purification of TGM2

The His6-tagged human TGM2 bacterial expression plasmid – pHis-hTG2 – was obtained from Addgene (Addgene plasmid # 100719) and was used to transform the *E. coli* strain BL21-Codon Plus (DE3)-RIPL (Agilent Technologies, 230280). Single colonies were picked from LB plates containing ampicillin, chloramphenicol and streptomycin, and were grown to early log phase (OD600 of 0.2-0.4) in LB medium in the presence of ampicillin at 37 ºC. The temperature then was reduced to 16 ºC and cultures were induced with 0.2 mM IPTG for overnight expression at 16 ºC. Bacteria were harvested at 4800g for 20 minutes, and pellets were resuspended in 5 volumes of ice-cold lysis buffer containing 50mM Tris pH 7.5, 500mM NaCl, 50mM imidazole, 1mM

bioRxiv preprint doi: https://doi.org/10.1101/2024.09.19.613951; this version posted September 24, 2024. The copyright holder for this preprint (which was not certified by peer review) is the author/funder, who has granted bioRxiv a license to display the preprint in perpetuity. It is made available under aCC-BY 4.0 International license.

PMSF and 1x cOmplete™ EDTA-free protease inhibitor cocktail (Sigma, 11836170001). Bacteria were lysed by sonication using a 5 second pulse, 5 second rest setting with 40% output, for a total of 20 minutes, on ice. Crude lysates were centrifuged at 25,000g for 30 minutes at 4 ºC. The supernatant were passed through a syringe tip 0.45-micron filter unit and applied to a His-Trap affinity column (Cytiva, 17524701) equilibrated with lysis buffer (excluding protease inhibitors) on an Akta Purifier (Cytiva). Following washes of the column with lysis buffer, salt concentration were gradually reduced to 150 mM NaCl. Bound proteins were eluted with a gradient of Imidazole from 50 mM to 500 mM and eluates were collected on a fraction collector. Fractions containing the peak protein elution were pooled together and applied to a HiPrep 26/10 desalting column, equilibrated with storage buffer containing 50mM Tris pH 7.5 and 150mM NaCl. Eluate was concentrated using an Amicon Ultra centrifugal concentrator (Sigma) and applied to an ENrich SEC-650 10x300 Column (Biorad), equilibrated with storage buffer for separation of impurities based upon size exclusion. Peak TGM2 fractions were pooled together and concentrated again.

Purification of histone H3

Recombinant human histone H3.2 was expressed in *E. coli* BL21 (DE3), extracted by guanidine hydrochloride and purified by flash reverse chromatography, as previously described (*48*). The purified histones were analyzed by RP-LC-ESI-MS (*48*).

Purification of CaMKII

Human γCaMKII wildtype or Q285A, or αCaMKII wildtype, coding sequences were cloned into a mammalian expression vector (pcDNA3.1) under a CMV promoter, along with N-terminal FLAG-HA and C-terminal HA epitope tags (pIM.081 and pIM.082). Expi293F cells (ThermoFisher Scientific, A14527) were grown in Expi293 expression medium and were

bioRxiv preprint doi: https://doi.org/10.1101/2024.09.19.613951; this version posted September 24, 2024. The copyright holder for this preprint (which was not certified by peer review) is the author/funder, who has granted bioRxiv a license to display the preprint in perpetuity. It is made available under aCC-BY 4.0 International license.

transiently transfected with the plasmid using Expifectamine transfection reagent, according to manufacturer's protocol (ThermoFisher Scientific, A14524). Cells were harvested 48-72 hours post-transfection by centrifugation at 500g, washed once with D-PBS, and then the cell pellet was resuspended in ice-cold lysis buffer containing 50mM Tris pH 7.5, 300mM NaCl, 1% Triton X-100, 1mM DTT, cOmplete™ EDTA-free protease inhibitor cocktail (Sigma, 11836170001). Lysates were incubated on ice for 15 min before centrifugation at 25,000g for 30 minutes at 4 ºC. The supernatant was passed through a syringe tip 0.45 micron filter unit and applied to ANTI-FLAG® M2 Affinity Gel (Millipore Sigma, A2220) equilibrated with lysis buffer, rotating at 4 ºC for 2 hours. Following affinity binding incubation, lysate/bead mixtures were transferred to a chromatography column and washed extensively with lysis buffer (containing up to 0.5M NaCl), followed by washes with 50mM Tris pH 7.5, 150mM NaCl. Bead bound proteins were eluted with 0.1M Glycine, pH 3.5 solution. Eluate pH was immediately neutralized by adding 1/20th volume of 1M Tris, pH 8.0. Eluate was then passed through desalting columns equilibrated with the storage buffer consisting of 50mM Tris, pH 7.5, 150 NaCl and concentrated using Amicon Ultra centrifugal concentrator (Sigma).

In vitro **TGM2 monoaminylation biochemical assays**

TGM2 monoaminylation assays were performed by incubating 3μg of H3, αCaMKII, βCaMKII (ab132961) or γCaMKII with TGM2 (0.3μg) in 30μL of enzymatic buffer (25 mM Tris, 10 mM CaCl2, 10 mM DTT, 10 mM KCl, 5 mM MDC/5-HT/DA/NE/His, pH 7.8) for 1hr at RT. The samples were then prepped and run on a BisTris polyacrylamide gel and imaged using UV light (e.g., MDC assays) or western blotting for histone H3 (e.g., *in vitro* probe-IP experiment).

In vitro **Kinase Assay**

bioRxiv preprint doi: https://doi.org/10.1101/2024.09.19.613951; this version posted September 24, 2024. The copyright holder for this preprint (which was not certified by peer review) is the author/funder, who has granted bioRxiv a license to display the preprint in perpetuity. It is made available under aCC-BY 4.0 International license.

CaMKII Reaction Buffer
40mM Tris, pH 7.4
ATP 100μM
20mM MgCl2
.01mg/mL BSA
50μM DTT
100 μM $CaCl_2$

Recombinant WT and Q285A h-γCaMKII reactions were performed using the ADP-Glo Kinase assay (Promega, V9101) according to the manufacturer's instructions. Briefly, serial dilutions of rγCaMKII and rγCaMKIIQ285A (from 2.6μg to 16ng, 0ng) were plated in triplicate in CaMKII reaction buffer and incubated at RT for 30 minutes. Equivolume of ADP-Glo reagent was added and incubated at RT for 40 minutes. Kinase Detection Reagent were then added to each well and incubated for an additional 30 minutes at RT. Following incubation of kinase detection reagent, luminosity was measured using a Cytation3 (Biotek).

Luciferase Assay

Both WT and Q285A hippocampal primary neurons were treated with pGF-CREB-mCMV-EF1α-Puro lentiviral particles (Systems Biosciences, TR202va-p) on DIV2. At DIV 14, cells were treated with 500nM DA for 1 hour. Pierce Firefly Luciferase Kit (Thermo, 16175) was then used according to manufactures protocol. Luminescence was measured via a Cytation3 (Biotek).

Immunoprecipitation of dopaminylated proteins using the Bio-CO probe

Low Salt Buffer (LSB)	150 mM KCl<br>1% NP40<br>20mM HEPES pH 7.4<br>10 mM MgCl2
High Salt Buffer (HSB)	500 mM KCl<br>1% NP40<br>20mM HEPES pH 7.4<br>10 mM MgCl2

bioRxiv preprint doi: https://doi.org/10.1101/2024.09.19.613951; this version posted September 24, 2024. The copyright holder for this preprint (which was not certified by peer review) is the author/funder, who has granted bioRxiv a license to display the preprint in perpetuity. It is made available under aCC-BY 4.0 International license.

Briefly, Dynabeads M280 streptavidin (Thermo #11206D) were aliquoted and washed 2x with LSB+5% bovine serum albumin (BSA), followed by resuspension of beads in 500 µL LSB+5% BSA. Bio-CO probe (or desthiobiotin for – probe conditions) at a concentration of 1mg/mL in 1:1 acetonitrile and water was added to the tube and rotated at 4 ºC for 1 hour. While incubating, tissue samples (VTA, NAc, mPFC or dHPC; 2 mm punches) were homogenized in LSB + cOmplete EDTA-free protease inhibitor cocktail (Sigma #11873580001) via wand sonication. 5mg/mL $NaIO_4$ was then added to all samples, and incubated for 10 minutes on ice, flicking halfway through. Following incubation of bead with probe/desthiobiotin, samples were washed 1x with LSB and resuspended in LSB. 1M DTT was then added to samples before the addition of the conjugated bead to sample. Samples were then rotated for 1 hour at 4 ºC before being washed 6x with 1mL HSB. Following HSB washes, samples were washed with 500µL 0.2M glycine, pH 3.5, and the glycine was discarded. Beads were then prepped for downstream analysis (proteomics or western blotting).

Bio-CO Probe Western Blotting and antibodies

Following Bio-CO probe IPs, beads were resuspended in TBS with LDS sample buffer (Invitrogen #NP0007) and sample reducing agent (Invitrogen #NP0004), and then heated at 98 ºC for 7 minutes. Samples were placed back onto the magnet and supernatant loaded into 4-12% BisTris polyacrylamide gels (BioRad #3450125) for electrophoresis, followed by transfer to nitrocellulose membranes. Nitrocelluose membranes were then blocked in 5% BSA in TBS + 0.1% Tween 20 (TBST) for 1 hour followed by incubation with primary antibodies overnight at 4 ºC. The following primary antibodies were used: mouse anti-Histone H3 (1:15,000, Abcam ab10799), anti-αCaMKII (1:1,000, CST 50049S, anti-βCaMKII (1:1,000, Abcam ab34703) or mouse anti-γCaMKII (1:5000, Abcam ab201966). The following day, membranes were washed in TBST 3x for 10 minutes and

bioRxiv preprint doi: https://doi.org/10.1101/2024.09.19.613951; this version posted September 24, 2024. The copyright holder for this preprint (which was not certified by peer review) is the author/funder, who has granted bioRxiv a license to display the preprint in perpetuity. It is made available under aCC-BY 4.0 International license.

incubated for 1 hour at RT in TBST+5%BSA with anti-Mouse Alex Fluor™ 647 (1:10,000, Invitrogen A-21242), anti-Mouse Alex Fluor™ 546 (1:10,000, Invitrogen A-21123), and/or anti-Rabbit Alex Fluor™ 647 (1:10,000, Invitrogen A-21246). This was followed by 3 washes in TBST for 10 minutes each before quantification using fluorescence via a ChemiDoc MP imaging system (Biorad). Densitometry was used to quantify protein bands using Image J Software (NIH).

Mass spectrometry

Proteins on magnetic beads underwent reduction and alkylation, followed by partial on-bead digestion with Trypsin (Promega) for 3 hours at room temperature. Supernatant was extracted, and proteins were re-digested with Lys-C (Wako/Fuji) and Trypsin overnight. Digestion reactions were stopped with neat Trifluoroacetic acid. Samples were Solid Phase Extracted (*49*), prior to being analyzed by LC-MS/MS. A 50 min or 70 min analytical gradient (typical 2% B/98%A to 32%B/68%A, A: 0.1% formic acid, B: 80% acetonitrile/0.1% formic acid) was used and peptides were measured using high resolution/high mass accuracy mass spectrometers (Q-Exactive, Fusion Ascend or Fusion Lumos, ThermoFisher Scientific). Generated Data Dependent Acquisition data were searched and quantified using ProteomeDiscoverer (ThermoFisher Scientific)/Mascot (MatrixScience) or MaxQuant v.1.6 or higher. Depending on the experiments, UniProt's mouse, rat or human databases were queried concatenated with Trypsin and Lys-C, as well as other potential contaminants (*50*). All quantitative data was generated as label free experiments. Analyses targeting specific peptides were designed as Parallel Reaction Monitoring (PRM) experiments and ion traces were extracted manually. Statistical analysis was carried out using Perseus v.2.0: prior to tests, signals were LOG2 transformed, possible contaminants were removed, and it was required that a protein must be measured in at least 2/3 of replicates in at least one sample group. Missing values were imputed.

bioRxiv preprint doi: https://doi.org/10.1101/2024.09.19.613951; this version posted September 24, 2024. The copyright holder for this preprint (which was not certified by peer review) is the author/funder, who has granted bioRxiv a license to display the preprint in perpetuity. It is made available under aCC-BY 4.0 International license.

Subcellular fractionations

Mouse dorsal hippocampus (dHPC) was homogenized in Buffer A (10mM HEPES (pH 7.9), 10 mM KCl, 1.5 mM $MgCl_2$, 0.34 M sucrose, 10% glycerol, and 1mM EDTA, and 1X protease inhibitor cocktail) using a dounce. Following transfer to a microcentrifuge tube, Triton-100X was added to a final concentration of 0.1%, and the samples were incubated on ice for 30 minutes before being centrifuged for 5 min at 1300g at 4 °C. The supernatant was decanted and reserved on ice. Nuclear pellets were resuspended with Buffer A to wash, and then spun in the centrifuge for 5 min at 1300g at 4 °C, before being decanted and supernatant discarded. Nuclear pellets were then resuspended in LSB (see above) with protease inhibitor cocktail. For experiments involving synaptosomal fractions, supernatant was then centrifuged at 20000g at 4 ºC for 10 minutes. Following centrifugation, supernatants were removed to a new tube (cytosolic fraction), and the crude synaptosomal pellet was resuspended in LSB. Crude synaptic and nuclear pellets were then sonicated to ensure homogeneity.

Western Blotting

Samples (10-20µg total protein) were loaded into BisTris polyacrylamide gels (Invitrogen or BioRad) for electrophoresis and then proteins were transferred to nitrocellulose membranes. Nitrocelluose membranes were then blocked in 5% BSA in TBS + 0.1% Tween 20 (TBST) for 1 hour followed by incubation with primary antibodies overnight at 4 ºC. The following primary antibodies were used: mouse anti-Histone H3 (1:15,000, Abcam ab10799), mouse anti-CaMKII gamma (1:5000, ab201966), anti-PSD95 (1:500, Abcam ab18258; note that 5% nonfat dry milk in TBST was used for blocking and incubation of this antibody based on manufacture recommendations), rabbit anti-CaMKIIβ (1:1000 Abcam ab34703), chicken anti-GAPDH

bioRxiv preprint doi: https://doi.org/10.1101/2024.09.19.613951; this version posted September 24, 2024. The copyright holder for this preprint (which was not certified by peer review) is the author/funder, who has granted bioRxiv a license to display the preprint in perpetuity. It is made available under aCC-BY 4.0 International license.

(1:10000Millipore AB2302), rabbit anti-pan-pCaMKIIT286 (1:1000 CST 12716S), Rabbit Anti-Sodium Potassium ATPase (1:10000 Abcam ab76020), rabbit anti-phospho-CREBS1333 (1:500 CST, 9198) or mouse anti-CREB (1:500 CST, 9104). The following day, membranes were washed in TBST 3 times for 10 minutes and incubated for 1 hour at RT in TBST+5%BSA with anti-Mouse Alex Fluor™ 546 (1:10,000, Invitrogen A-21123), anti-Rabbit Alex Fluor™ 647 (1:10000, A-21246) or anti-Chicken Alexafluor 488 (1:10000, Invitrogen A-11039). This was followed by 3 washes in TBST for 10 minutes each before quantification using fluorescence via a ChemiDoc MP imaging system (Biorad). Densitometry was used to quantify protein bands using Image J Software (NIH), and proteins were normalized to appropriate loading controls for fraction.

Co-immunoprecipitations

Low Salt CoIP Buffer(LSCB)	150 mM KCl<br>0.5% NP40<br>20mM HEPES pH 7.4<br>10 mM MgCl2
High Salt CoIP Buffer(HSCB)	350 mM KCl<br>0.5% NP40<br>20mM HEPES pH 7.4<br>10 mM MgCl2

Dynabeads M280 Sheep-Anti Mouse IgG beads are washed with LSCB before being resuspended in LSCB+5% BSA with mouse anti-CaMKII gamma (Abcam ab201966) and rotated at 4 ºC for 1 hour. Conjugated beads are then washed and resuspended in LSCB and added to mouse dHPC homogenized in LSCB. Sample-bead mixture incubated via rotation at 4 ºC for 4 hour. Following incubation, supernatants were removed and discarded, and samples were washed with HSCB 6x. Samples are then resuspended in TBS and prepped for western blotting.

Single-nuclei RNA-seq

bioRxiv preprint doi: https://doi.org/10.1101/2024.09.19.613951; this version posted September 24, 2024. The copyright holder for this preprint (which was not certified by peer review) is the author/funder, who has granted bioRxiv a license to display the preprint in perpetuity. It is made available under aCC-BY 4.0 International license.

Nuclei isolation and library preparation

Single-nuclei RNA-sequencing was performed on four 3mm dorsal hippocampal tissue punches from 8-week-old wildtype *vs.* γCaMKIIQ285A mutant mice. Nuclei were isolated using a modified version of the Matevossian and Akbarian sucrose density gradient isolation protocol (*51*). Briefly, tissues were thawed in 1 mL of lysis buffer (0.32 M Sucrose, 5 mM $CaCl_2$, 3 mM magnesium acetate, 0.1 mM EDTA, 10 mM Tris-HCl pH 8, 1 mM DTT, 0.1% Triton X-100) with 50 µL of 25U/mL RNase inhibitor (Takara, cat#2313B) for 2 minutes in a 1 mL dounce homogenizer (Wheaton, cat# 357538). Tissues were gently homogenized with ~20 strokes using a tight homogenizer. An additional 1 mL of lysis buffer was added to the homogenizer, another 10 strokes were performed and the 2 mL homogenates were transferred to 15 mL Open-Top Thinwall Polypropylene Tubes 16 x 95 mm (Beckman, cat# 361707). The homogenizers and douncers were washed with an additional 2 mL of lysis buffer, which was added to the Polypropylene Tube for a total of 4 mL of tissue homogenates. Homogenates were carefully underlaid with 9 mL of sucrose solution (1.8 M sucrose, 3 mM magnesium acetate, 1 mM DTT, 10 mM Tris-HCl, pH 8) and ultracentrifuged in a Sorvall™ WX+ at 24,000 rpm for 1 hr at 4 °C.

Following centrifugation, supernatants and the debris interphases were gently aspirated, resuspended in 1mL of resuspension buffer [0.02% bovine serum albumin in DPBS 25 µl of 25U/mL RNase inhibitor (Takara, cat#2313B)] and incubated on ice for 10 minutes. Resuspensions were filtered through a 35 µm nylon mesh filter (Corning, cat#352235) into a 1.5mL RNase/DNase-free microcentrifuge tube and centrifuged at 2,600 x g for 10 minutes at 4 °C. Supernatants were removed and nuclear pellets were resuspended in 200 uL of resuspension buffer. 10 uL of nuclei were stained with Trypan Blue, and the quality and quantity of nuclei were assessed using a Countess 3 Automated Cell Counter.

bioRxiv preprint doi: https://doi.org/10.1101/2024.09.19.613951; this version posted September 24, 2024. The copyright holder for this preprint (which was not certified by peer review) is the author/funder, who has granted bioRxiv a license to display the preprint in perpetuity. It is made available under aCC-BY 4.0 International license.

Nuclear suspensions were then loaded onto a Chromium Single cell 3′ chip, version 3 (10X Genomics) and processed according to manufacturer's protocol with a target of 8,000 cells. Single-nuclei libraries were prepared as per the 10X Chromium Next GEM Single Cell 3' v3.1 (Dual Index) Protocol (CG000315 Rev A) being pooled onto a single 10B 100 Cycle Flowcell and sequenced using the Illumina NovaSeq X Plus system to obtain paired-end 2 × 100 bp reads.

Data analysis

FastQ files were processed with the 10X Genomics Cell Ranger single-cell analysis pipeline (Cell Ranger v7.1.0) to demultiplex reads, align against the mouse genome (mm10-2020-A), deduplicate reads, and extract the gene expression matrix. Only confidently mapped, non-PCR duplicated reads with valid single-cell barcodes and UMIs were be used to generate gene-barcode matrices for further analysis. For each sample, we used the SoupX (*52*) (v1.6.2) package to automatically calculate the contamination fraction from the raw and filtered Cell Ranger output feature matrices, and then to remove background contamination from the count matrix. The Seurat pipeline (*53*) (v. 5.1.0) was used to carry out downstream analyses on the adjusted count matrices as Seurat objects. First, quality control metrics were calculated per sample, and nuclei were filtered based on the following conditions: nFeature_RNA > 250 & nCount_RNA > 1000 & nCount_RNA < 110000 & percent.mt < 5 & percent.ribo < 5. Individual sample counts were normalized with *NormalizeData*, top variable genes were identified with *FindVariableFeatures* (using the "vst" method), and genes were centered and scaled with *ScaleData,* followed by *RunPCA* dimensionality reduction and *RunUMAP*. Individual samples were then integrated with Harmony (*54*) (v1.2.0). Next, distinct cell types were identified using Louvain (*55*) clustering and visualized by UMAP (*56*). Nearest neighbors were identified, and clusters were identified by a shared nearest

bioRxiv preprint doi: https://doi.org/10.1101/2024.09.19.613951; this version posted September 24, 2024. The copyright holder for this preprint (which was not certified by peer review) is the author/funder, who has granted bioRxiv a license to display the preprint in perpetuity. It is made available under aCC-BY 4.0 International license.

neighbor (SNN) modularity optimization based algorithm (*FindClusters).* Cell clusters were annotated as major hippocampal cell-types by expression of known cell-type markers and through Seurat's reference mapping capability with a previously published hippocampal single-cell sequencing anchor dataset from the Allen Brain Map resource (*57*).

Astrocytes: *Aldh1l1, Gfap, Fzd2*

Microglia: *C1qc*

Oligodendrocytes: *Myrf, Mag, Mog*

OPCs: *Pdgfra*

Endothelial Cells: *Flt4 (58)*

Pericytes: *Vtn* (*59*)

Nueral progenitor Cells (*60*): *Neurod1, Ccnd2*

Gabaergic: *Gad2*

Neural Stem Cells (*60*): *Id4, Sox2, Fgfr3*

CA1(*59*): *Wfs1, Ndst4*

CA3 (*57, 59*): *Iyd, Cpne4*

Subiculum (*57*): *Tshz2*

For differential expression testing, we used Seurat's pseudobulk capability with *AggregateExpression* to sum gene counts from all the cells from the same sample for each cell type cluster. We then used DESeq2 at the sample level to perform differential expression testing with multiple hypothesis testing adjusted using FDR<5%. The clusterProfiler package (v 2.1.6) was implemented for pathway analysis of identified DEGs. We used the fgsea package (*61*) (v 1.30.0) to run gene set enrichment analysis on the output from pseudobulk differential expression testing.

bioRxiv preprint doi: https://doi.org/10.1101/2024.09.19.613951; this version posted September 24, 2024. The copyright holder for this preprint (which was not certified by peer review) is the author/funder, who has granted bioRxiv a license to display the preprint in perpetuity. It is made available under aCC-BY 4.0 International license.

The hdWGCNA (*62*) package (v. 0.3.03) was utilized to identify and visualize co-expression gene network modules with a soft power threshold of 6 (threshold identified via *TestSoftPowers*), as well as to visualize module enrichment and expression of the modules across cell-types. The hdWGCNA package was also used to calculate the correlation between modules by annotated cell-type and the trait of mutation (γCaMKIIQ285A) by Spearman's rank correlation coefficients, with Student's *t*-tests and a p value of < 0.05 being considered statistically significant. Finally, we used the EnrichR function within hdWGCNA to perform pathway analysis on the genes within each module.

Ex vivo whole-cell patch-clamp recordings

4-8 week old male transgenic (Q285A) and wild-type littermate mice were anesthetized using isoflurane. Brains were rapidly extracted, and coronal sections (250 microns) were prepared using a Compresstome (Precisionary Instruments Inc.) in cold (0-4 ºC) sucrose-based artificial cerebrospinal fluid (SB-aCSF) containing 87 mM NaCl, 2.5 mM KCl, 1.25 mM NaH2PO4, 4 mM MgCl2, 23 mM NaHCO3, 75 mM Sucrose, 25 mM Glucose. After recovery for 60 minutes at 32 °C in oxygenated (95% CO2 / 5% O2) aCSF containing 130 mM NaCl, 2.5 mM KCl, 1.2 mM NaH2PO4, 2.4 mM CaCl2, 1.2 mM MgCl2, 23 mM NaHCO3, 11 mM Glucose, slices were kept in the same medium at RT for the rest of the day and individually transferred to a recording chamber continuously perfused at 2-3 mL/min with oxygenated aCSF. Patch pipettes (5-7 MΩ) were pulled from thin wall borosilicate glass using a micropipette puller (Sutter Instruments) and filled with a K-Gluconate-based intra-pipette solution containing 116 mM KGlu, 20 mM HEPES, 0.5 mM EGTA, 6 mM KCl, 2 mM NaCl, 4 mM ATP, 0.3 mM GTP (pH 7.2). Cells were visualized using an upright microscope with an IR-DIC lens and illuminated with a white light source (Olympus for Scientifica) and using the MicroManager v2.0 software (https://micromanager.org/).

bioRxiv preprint doi: https://doi.org/10.1101/2024.09.19.613951; this version posted September 24, 2024. The copyright holder for this preprint (which was not certified by peer review) is the author/funder, who has granted bioRxiv a license to display the preprint in perpetuity. It is made available under aCC-BY 4.0 International license.

All recordings were made in dorsal CA1. Excitability was measured in current-clamp mode by injecting incremental steps of current (0–300 pA, +20 pA at each step). Whole-cell recordings were performed using a patch-clamp amplifier (Axoclamp 200B, Molecular Devices) connected to a Digidata 1550 LowNoise acquisition system (Molecular Devices). Signals were low pass filtered (Bessel, 2 kHz) and collected at 10 kHz using Axon pCLAMP 11 Software Suite (Molecular Devices). Electrophysiological recordings were extracted using Clampfit (Molecular Devices). All groups were counterbalanced by days of recording and all recordings were performed blind to experimental conditions.

Contextual fear conditioning

WT and Q285A male mouse littermates were habituated to the testing chamber for 15 minutes. The following day, mice were trained over 5 conditioning trials, each consisting of pseudo-random interval, with a 2.0s, 0.9mA foot shock. Testing for conditioned fear response (freezing) occurred 24 hour following training. Mice were placed into the conditioned context for 5 minutes, and total time freezing was recorded (VFC chamber, Med Associates). Freezing is expressed as a percentage of the total test time.

Viral transduction

28 days before contextual fear conditioning, training animals were anaesthetized via ketamine/xylazine (10/1 mg/kg) i.p., positioned in a stereotaxic frame (Kopf instruments), and 0.5µl of viral construct (lenti-RFP *vs.* lenti-γCaMKII-RFP) was infused bilaterally into dHPC using the following coordinates from bregma; anterior-posterior (AP) -2.2, medial-lateral (ML) ±1.3, dorsal-ventral (DV) -1.6. Mice received meloxicam (1 mg/kg) s.c. and topical antibiotic

bioRxiv preprint doi: https://doi.org/10.1101/2024.09.19.613951; this version posted September 24, 2024. The copyright holder for this preprint (which was not certified by peer review) is the author/funder, who has granted bioRxiv a license to display the preprint in perpetuity. It is made available under aCC-BY 4.0 International license.

treatments for 3 days following surgery. Behavioral testing and transduced tissue collection occurred at least 28 days following transduction to ensure maximal infection.

Immunohistochemistry/immunofluroscence

Mice were anesthetized with isoflurane and perfused with cold 1X PBS, followed by 4% PFA. The brains were post-fixed overnight in 4% PFA and then placed in a 30% sucrose/PBS solution for two days. After a 1x PBS wash, the brains were embedded in Tissue-Tek® O.C.T. Compound (Sakura, #4583) and sectioned at a thickness of 40 µm using a cryostat (Leica CM3050-S). Serial sections were collected from the dHP and stored at 4°C in 1X PBS with 0.01% sodium azide until immunofluorescence processing.

For immunofluorescence, brain sections were washed three times in 1X PBS for 10 minutes each at room temperature (RT). The sections were then permeabilized with 0.2% Triton X in PBS for 30 minutes, followed by a 1-hour incubation at RT in blocking buffer (0.3% Triton X, 3% normal donkey serum, 1X PBS). To amplify the fluorescent signal of the viral constructs, the sections were incubated overnight at 4°C with mouse anti-RFP primary antibodies (ThermoFisher, catalogue #MA5-15257, 1:500). The following day, after three washes in 1X PBS, the sections were incubated for 2 hours at RT on a slow shaker with secondary antibodies (donkey anti-mouse AlexaFluor680 – ThermoFisher A-21109; 1:1000) in blocking solution. Afterward, the sections were washed three times in 1X PBS for 10 minutes each and incubated with DAPI (1:10000, Thermo Scientific 62248) for 5 minutes. Finally, the sections were washed in 1X PBS and mounted on charged thermofrost slides using ProLong Gold Antifade Mountant (ThermoFisher, Cat. No. P36934).

bioRxiv preprint doi: https://doi.org/10.1101/2024.09.19.613951; this version posted September 24, 2024. The copyright holder for this preprint (which was not certified by peer review) is the author/funder, who has granted bioRxiv a license to display the preprint in perpetuity. It is made available under aCC-BY 4.0 International license.

Digital images were captured using a confocal microscope (Zeiss LSM 780, upright) with Zen Black software. Images of the dHP were acquired at 20x magnification using an air objective (Plan-Apochromat 20x/0.8), with 4x3 tiled images. Eight consecutive acquisitions were averaged for each image at a bit depth of 16 bits. The fluorescent signal in the representative images was visualized as follows: RFP with excitation (Ex) at 561 nm and emission (Em) at 632 nm, and DAPI with excitation (Ex) at 405 nm and emission (Em) at 498 nm. The images were saved in .tiff or. czi format. In the representative images, the scale bar is set to 500 µm.

Statistics

Statistical analyses were performed using Prism GraphPad software. For experiments comparing only two conditions, two-tailed Student's t tests were performed. For experiments involving more than two conditions, one-way or two-way ANOVAs were performed with subsequent *post hoc* analyses. Sequencing-based statistical analyses are described above. In biochemical, physiological, behavioral and snRNA-seq analyses, all animals used were included as separate *n*s (i.e., samples were not pooled). Significance was determined at $p \leq 0.05$. All bar/dot and line plot data are represented as mean ± SEM.

bioRxiv preprint doi: https://doi.org/10.1101/2024.09.19.613951; this version posted September 24, 2024. The copyright holder for this preprint (which was not certified by peer review) is the author/funder, who has granted bioRxiv a license to display the preprint in perpetuity. It is made available under aCC-BY 4.0 International license.

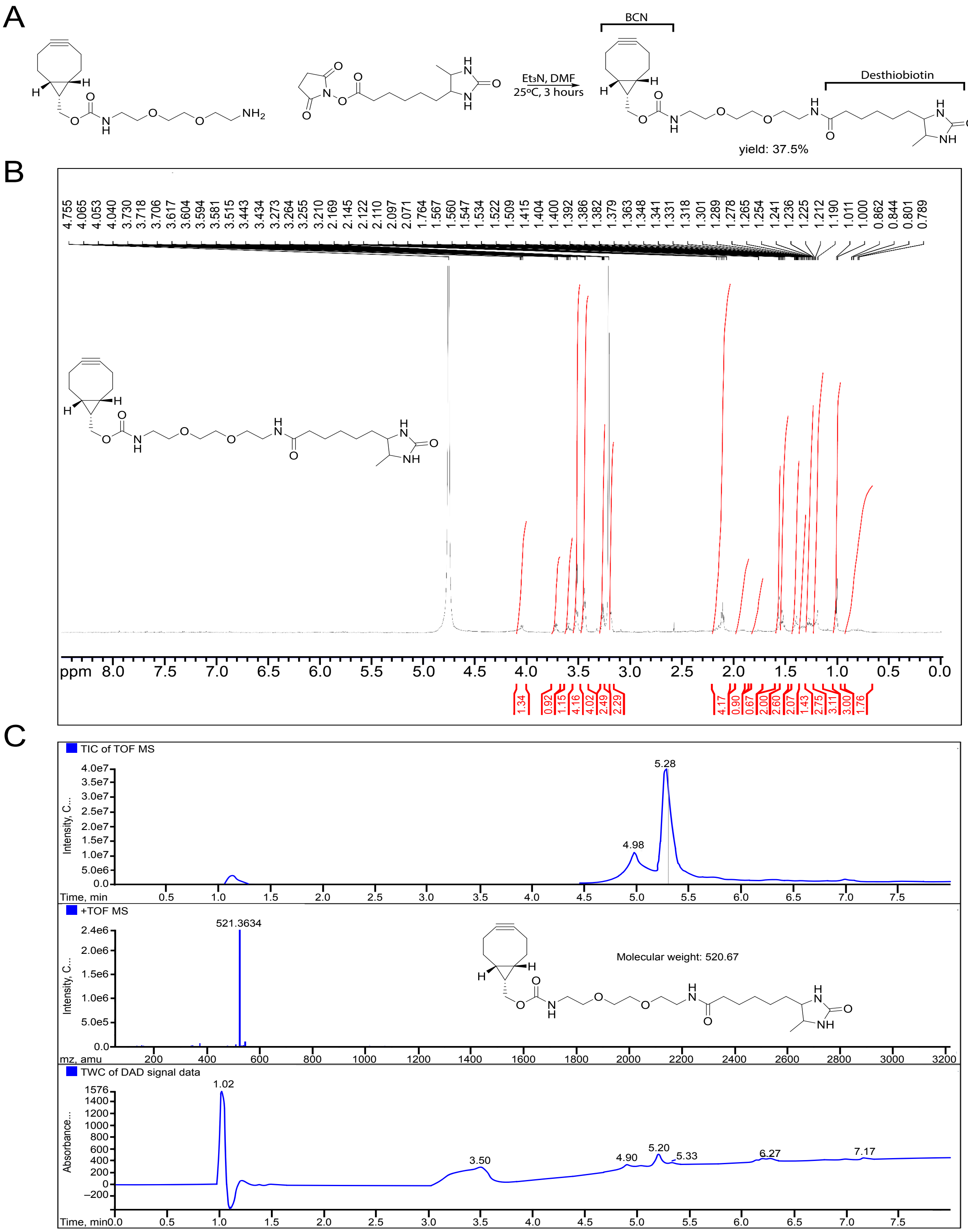

Figure S1: Synthesis and chemical validation of the Bio-CO probe.

A) Chemical scheme for catecholaminylation probe synthesis: Bio-CO.
B) ^{1}H NMR spectroscopy validation of Bio-Co probe.

bioRxiv preprint doi: https://doi.org/10.1101/2024.09.19.613951; this version posted September 24, 2024. The copyright holder for this preprint (which was not certified by peer review) is the author/funder, who has granted bioRxiv a license to display the preprint in perpetuity. It is made available under aCC-BY 4.0 International license.

C) LC-MS validation of Bio-CO probe.

bioRxiv preprint doi: https://doi.org/10.1101/2024.09.19.613951; this version posted September 24, 2024. The copyright holder for this preprint (which was not certified by peer review) is the author/funder, who has granted bioRxiv a license to display the preprint in perpetuity. It is made available under aCC-BY 4.0 International license.

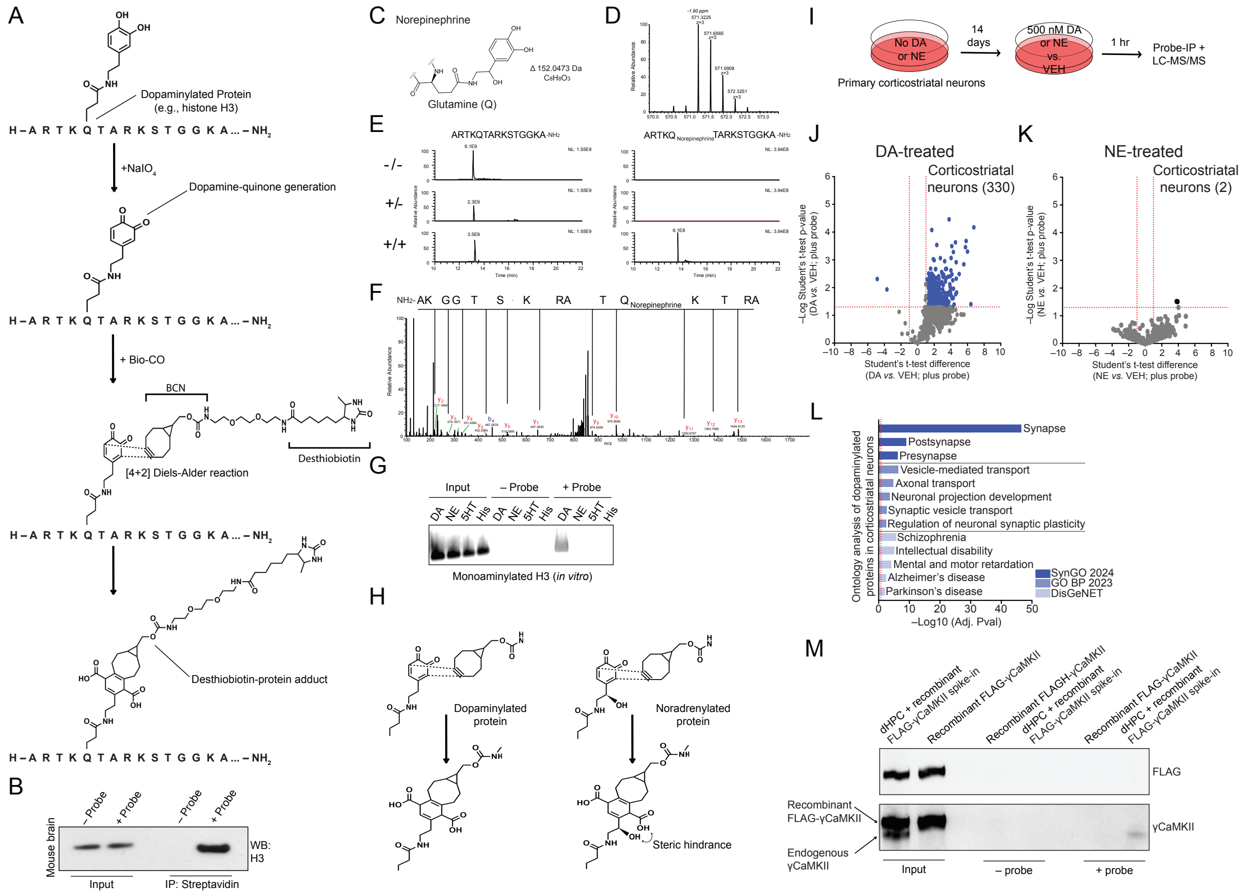

Figure S2: *in vitro, in cellulo* and *in vivo* validations of the Bio-CO catecholaminylation probe.

A) Schematic of catecholaminylation probe-mediated bioorthogonal labeling of endogenous dopaminylated proteins.

B) Catecholaminylation probe-mediated bioorthogonal labeling of endogenously dopaminylated histone H3 from mouse brain. Inputs = 1%; Streptavidin IPs were performed for – *vs.* + probe conditions, followed by blotting for H3.

C) Proposed structure of noradrenylated glutamine.

D) High resolution/high mass accuracy mass spectrum of the noradrenylated H3 tail peptide (ARTKQTARKSTGGKA-NH_3; +NE/+TGM2 condition).

E) Ion traces of the amidated H3 tail peptide with (right panels) and without (left panels) noradrenylated glutamine. Top, middle and bottom panels show signals measured under –/–, –/+ and +/+ (NE/TGM2) conditions, respectively.

F) Tandem MS of the glutamine 5 noradrenylated H3 tail peptide (+NE/+TGM2 condition). Selected fragment ions (y and b) are labeled. Vertical lines within the peptide sequence are used to highlight simplified peptide fragmentation.

G) Catecholaminylation probe-mediated bioorthogonal labeling of dopaminylated – but not serotonylated, histaminylated or noradrenylated – histone H3 *in vitro* following TGM2-dependent transglutamination. Inputs = 1%; Streptavidin IPs were performed for – *vs.* + probe conditions, followed by blotting for H3.

bioRxiv preprint doi: https://doi.org/10.1101/2024.09.19.613951; this version posted September 24, 2024. The copyright holder for this preprint (which was not certified by peer review) is the author/funder, who has granted bioRxiv a license to display the preprint in perpetuity. It is made available under aCC-BY 4.0 International license.

H) Chemical rationale for diminished recognition of noradrenylated proteins by the catecholaminylation probe under the specific reaction conditions used in this study (e.g., steric hindrance).
I) Schematic of monoamine (dopamine/DA or norepinephrine/NE) treatments of primary cultured mouse corticostriatal neurons, followed by probe IP-MS.
J) Volcano plot of MS-identified proteins enriched by the catecholaminylation probe following treatments with DA *vs.* vehicle/VEH (Student's t-test corrected for multiple comparisons; + probe: DA *vs.* VEH: n = 1%; $p<0.05$, fold-change >1).
K) Volcano plot of MS-identified proteins enriched by the catecholaminylation probe following treatments with NE *vs.* vehicle/VEH (Student's t-test corrected for multiple comparisons; + probe: NE *vs.* VEH: n = 1%; $p<0.05$, fold-change >1).
L) Ontology analysis (SynGO 2024, GO BP 2023, DisGeNET) of MS-identified proteins enriched by the catecholaminylation probe following treatments with DA (FDR<0.05; Benjamini-Hochberg).
M) Control experiment demonstrating that endogenous γCaMKII (dHPC), but not spike-in recombinant γCaMKII (purified using Expi293 cells, which lack endogenous monoaminylation owing to a lack of TGM2 expression) can be efficiency IP'd using the catecholaminylation probe.

See **Figure S8** for uncropped blot images.

bioRxiv preprint doi: https://doi.org/10.1101/2024.09.19.613951; this version posted September 24, 2024. The copyright holder for this preprint (which was not certified by peer review) is the author/funder, who has granted bioRxiv a license to display the preprint in perpetuity. It is made available under aCC-BY 4.0 International license.

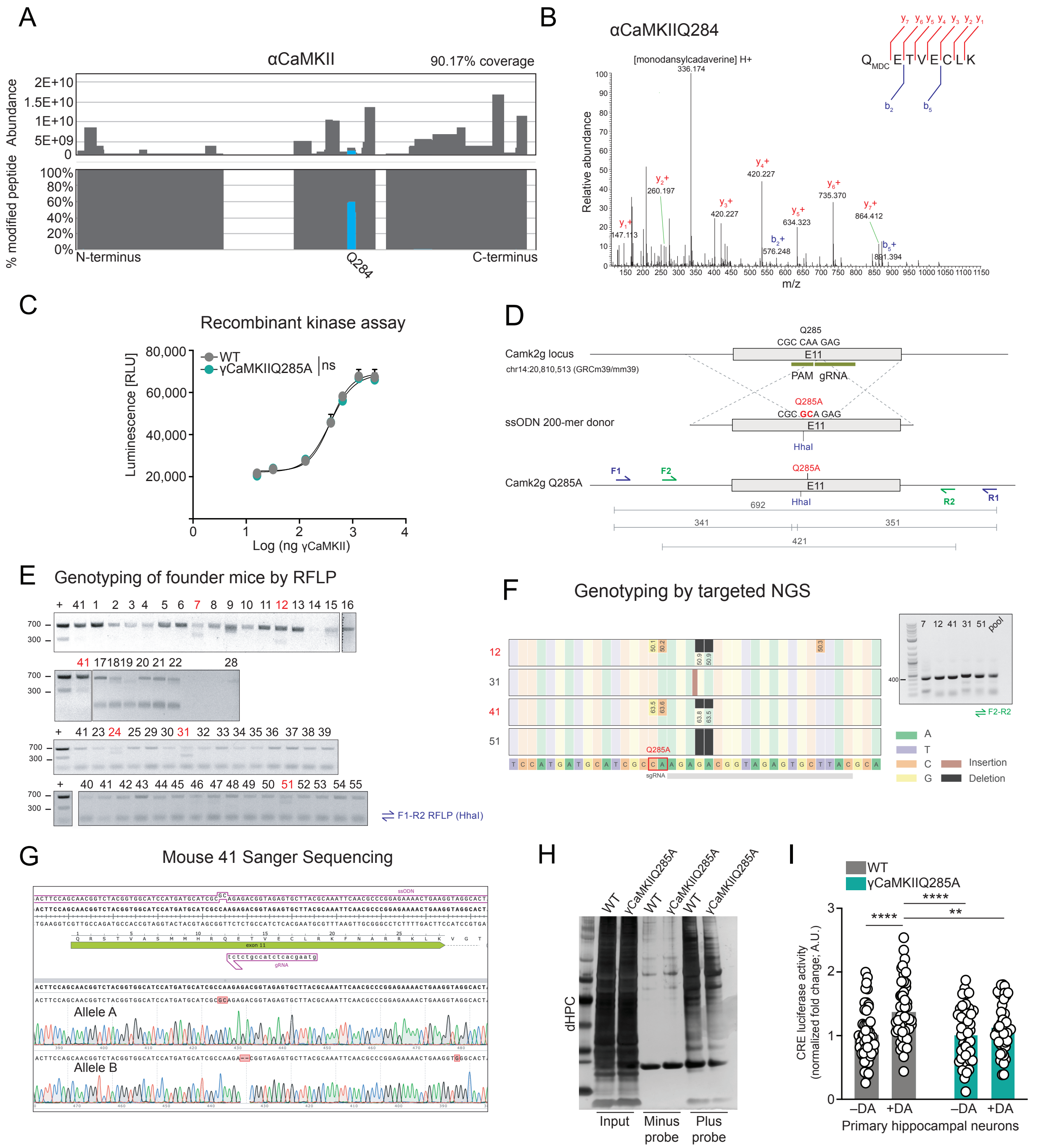

Figure S3: Generation of γCaMKIIQ285 knock-in mutant mice.

A) Lego plot indicating the abundance and % of MDC modified peptide in αCaMKII (90.17% coverage) following TGM2-mediated transglutamination. Note that only αCaMKIIQ284 was observed to be modified by MDC.

B) MS spectra for MDCylation of αCaMKII at glutamine 284. Y+ and b+ ions are annotated in red and blue, respectively.

C) *in vitro* recombinant γCaMKII kinase activity assay, demonstrating that the Q285A mutation does not impact γCaMKII's intrinsic kinase activity (n =3/γCaMKII concentration). The curves

bioRxiv preprint doi: https://doi.org/10.1101/2024.09.19.613951; this version posted September 24, 2024. The copyright holder for this preprint (which was not certified by peer review) is the author/funder, who has granted bioRxiv a license to display the preprint in perpetuity. It is made available under aCC-BY 4.0 International license.

were not found to be statically different via nonlinear fit regression analysis. Data presented as mean ± SEM.

D) Schematic of gene editing approach. Guide RNA and PAM sequences are indicated in green, the 200-mer ssODN donor used to mediate HDR contained the CA>GC change highlighted in red. The CA>GC generated the Q285A mutation ,and the generation of HhaI recognition site was used for the screening. Primers used for the screening are indicated in purple and green.

E) Genotyping of founder mice by restriction fragment length polymorphism (RFLP). Tail genomic DNA was amplified using F1 and R1 primers, followed by RFLP with HhaI. The expected bands (341 and 351 bp) for HDR events are detected in mice indicated in red. Positive control (+) is a spike-in of dsDNA containing the entire locus sequence spanning across F1 and R1 primers and containing the CA>GC change.

F) Genotyping by targeted next generation sequencing. Mice 12, 31, 41 and 51 were selected for further validation. The amplicon generated using F2 and R2 primers of 421 bp was subjected to Illumina amplicon sequencing and analyzed using the CRISPResso tool. Mice 12 and 41 showed the expected HDR CA>GC change, but also a GA deletion in about half of the reads.

G) Mouse 41 Sanger sequencing. The F2/R2 amplicon was cloned into a sequencing vector (pCR Blunt II-TOPO) and plasmids derived from single *E. coli* clones were subjected to Sanger sequencing, revealing that mouse 41 carried the CA>GC change and the GA deletion on different alleles. Mouse 41 was therefore used as the founder for the γCaMKIIQ285A mouse colony.

H) Silver stain of catecholaminylation probe-mediated bioorthogonal labeling and enrichment of proteins from dHPC of WT *vs.* γCaMKIIQ285A mice, indicating that the Q285A mutation does not globally alter protein domainylation. Inputs =1%; Streptavidin IPs were performed for – *vs.* + probe conditions.

I) CRE luciferase assay in WT *vs.* γCaMKIIQ285A primary cultured hippocampal neurons treated with dopamine *vs.* vehicle (VEH: WT *vs.* γCaMKIIQ285A, n = 47/genotype; DA: WT *vs.* γCaMKIIQ285A, n = 48 and 46/genotype, respectively). Analyzed by two-way ANOVA – main effects of treatment ($F_{1,184}$ = 14.90, ***p = 0.0002) and genotype ($F_{1,184}$ = 3.886, *p = 0.0502), with significant post hoc analyses (uncorrected Fisher's LSD) for: WT VEH *vs.* WT DA (****p<0.0001), Q285A VEH *vs.* WT DA (****p<0.0001) and WT DA *vs.* Q285A DA (**p = 0.0072).

All line/bar plots presented as mean ± SEM. See **Figure S8** for uncropped blot images.

bioRxiv preprint doi: https://doi.org/10.1101/2024.09.19.613951; this version posted September 24, 2024. The copyright holder for this preprint (which was not certified by peer review) is the author/funder, who has granted bioRxiv a license to display the preprint in perpetuity. It is made available under aCC-BY 4.0 International license.

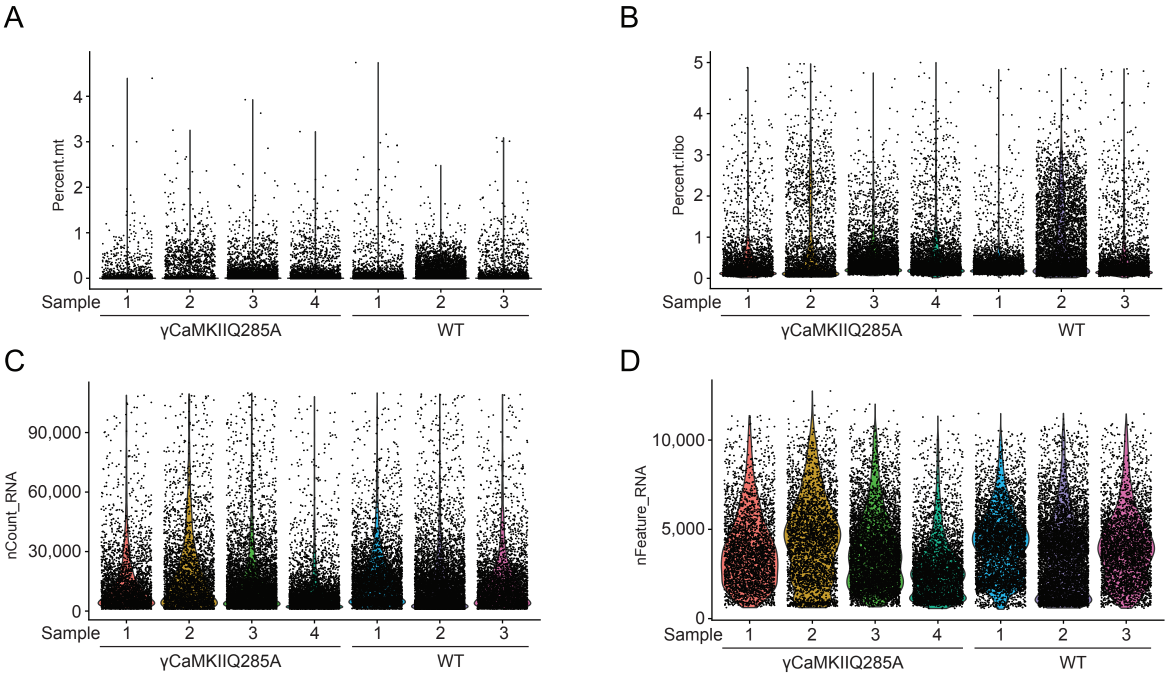

Figure S4: snRNA-seq quality control assessments.

A) Violin plot showing the % of mitochondrial genes by sample (WT: *n* = 3 *vs.* γCaMKIIQ285A: *n* = 4) in snRNA-seq dataset after filtering.
B) Violin plot showing the % of ribosomal RNA by sample (WT: *n* = 3 *vs.* γCaMKIIQ285A: *n* = 4) in snRNA-seq dataset after filtering.
C) Violin plot showing the number of UMI by sample (WT: *n* = 3 *vs.* γCaMKIIQ285A: *n* = 4) in snRNA-seq dataset after filtering.
D) Violin plot showing the number of detected genes by sample (WT: *n* = 3 *vs.* γCaMKIIQ285A: *n* = 4) in snRNA-seq dataset after filtering.

bioRxiv preprint doi: https://doi.org/10.1101/2024.09.19.613951; this version posted September 24, 2024. The copyright holder for this preprint (which was not certified by peer review) is the author/funder, who has granted bioRxiv a license to display the preprint in perpetuity. It is made available under aCC-BY 4.0 International license.

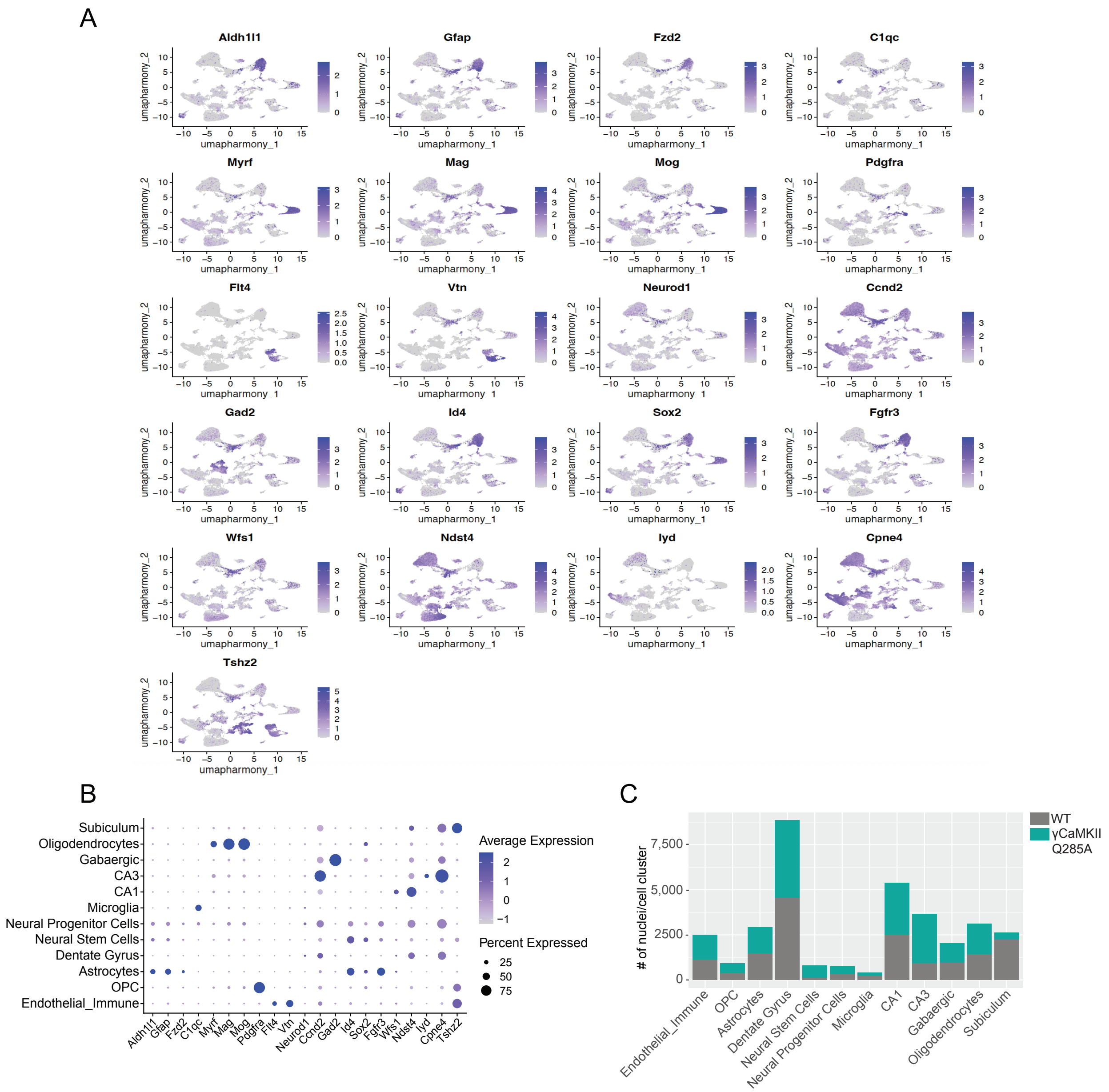

Figure S5: Cell cluster annotation from snRNA-seq dataset.

A) Unsupervised clustering of snRNA-seq dataset (WT: *n* = 3 *vs.* γCaMKIIQ285A: *n* = 4) with cells colored by cell-type specific marker genes (see **Figure 2A** for cell cluster annotation by cell-type).

B) Bubble plot indicating the average expression and % of cells expressing cell-type specific marker genes across annotated cell clusters (determined using unsupervised clustering).

C) Stacked bar plot indicating the # of nuclei called per cell annotated cell cluster, separated by genotype (WT: *n* = 3 *vs.* γCaMKIIQ285A: *n* = 4).

bioRxiv preprint doi: https://doi.org/10.1101/2024.09.19.613951; this version posted September 24, 2024. The copyright holder for this preprint (which was not certified by peer review) is the author/funder, who has granted bioRxiv a license to display the preprint in perpetuity. It is made available under aCC-BY 4.0 International license.

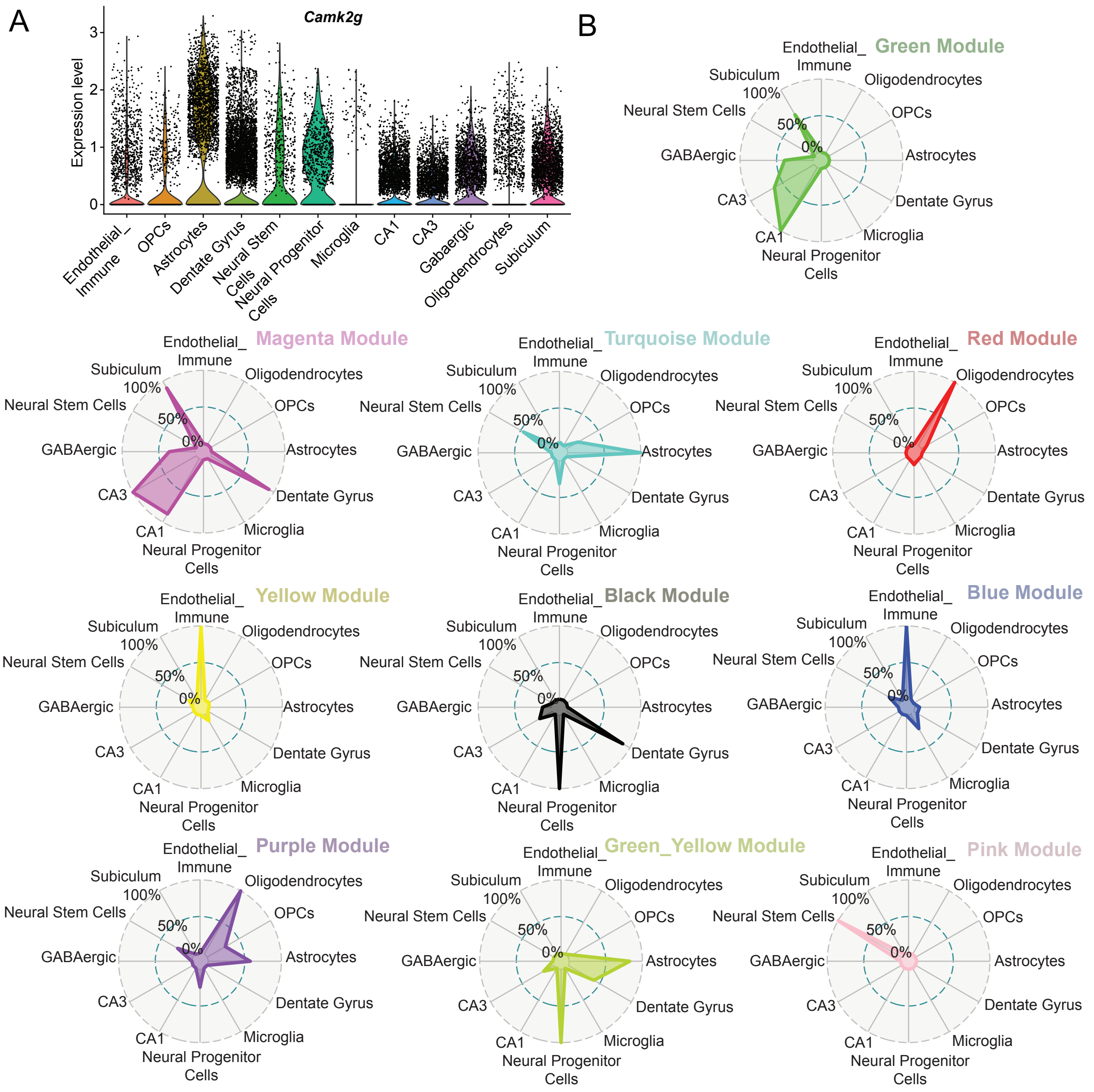

Figure S6: *γCaMKII* expression across cell clusters and WGCNA-identified module enrichment by cell cluster.

A) *γCaMKII* RNA expression across annotated cell clusters in snRNA-seq dataset (WT: $n = 3$ *vs.* γCaMKIIQ285A: $n = 4$). Note that *γCaMKII* expression was not found to differ by genotype.
B) Radar plots of WGCNA-identified module enrichment by annotated cell cluster (WT: $n = 3$ *vs.* γCaMKIIQ285A: $n = 4$). % enrichment of module genes by cell cluster is noted.

bioRxiv preprint doi: https://doi.org/10.1101/2024.09.19.613951; this version posted September 24, 2024. The copyright holder for this preprint (which was not certified by peer review) is the author/funder, who has granted bioRxiv a license to display the preprint in perpetuity. It is made available under aCC-BY 4.0 International license.

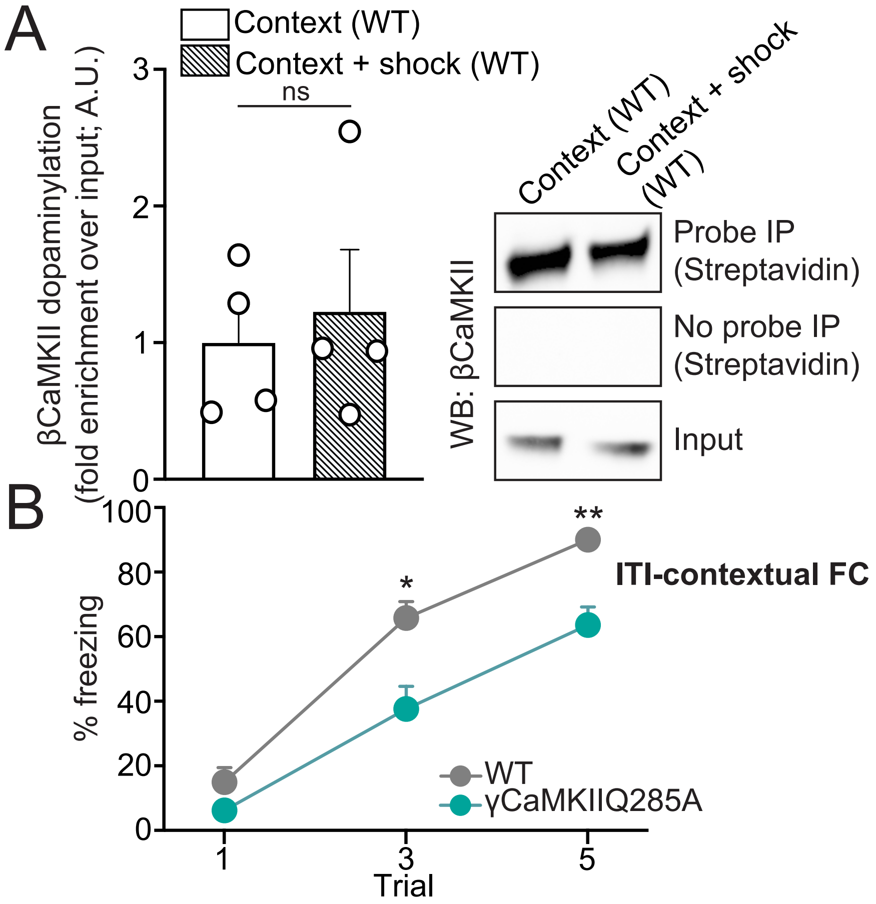

Figure S7: Contextual fear conditioning-induced dopaminylation is specific to γCaMKII and contributes to contextual learning in addition to contextual memory.

A) Bio-CO probe-mediated bioorthogonal labeling of dopaminylated βCaMKII from dHPC of wildtype mice + (context + shock) *vs.* – (context alone) contextual fear conditioning training (1 hr post-training). Streptavidin IPs were performed for – *vs.* + probe conditions and βCaMKII levels were normalized to respective inputs (1%). n = 4/genotype – significance determined by unpaired Student's t-test (t_6 = 0.4300, p = 0.6822).
B) Acquisition of contextual fear conditioning (average across inter-training interval/ITI trials) in wildtype *vs.* γCaMKIIQ285A mutant mice. Significance determined by repeated measures two-way ANOVA (main effect of trial x genotype; $F_{2,26}$ = 4.302, *p = 0.0243), followed by post hoc analysis (Sidak's MC test: *p = 0.0225, **p = 0.0076).

All bar/line plots presented as mean ± SEM. See **Figure S8** for uncropped blot images.

bioRxiv preprint doi: https://doi.org/10.1101/2024.09.19.613951; this version posted September 24, 2024. The copyright holder for this preprint (which was not certified by peer review) is the author/funder, who has granted bioRxiv a license to display the preprint in perpetuity. It is made available under aCC-BY 4.0 International license.

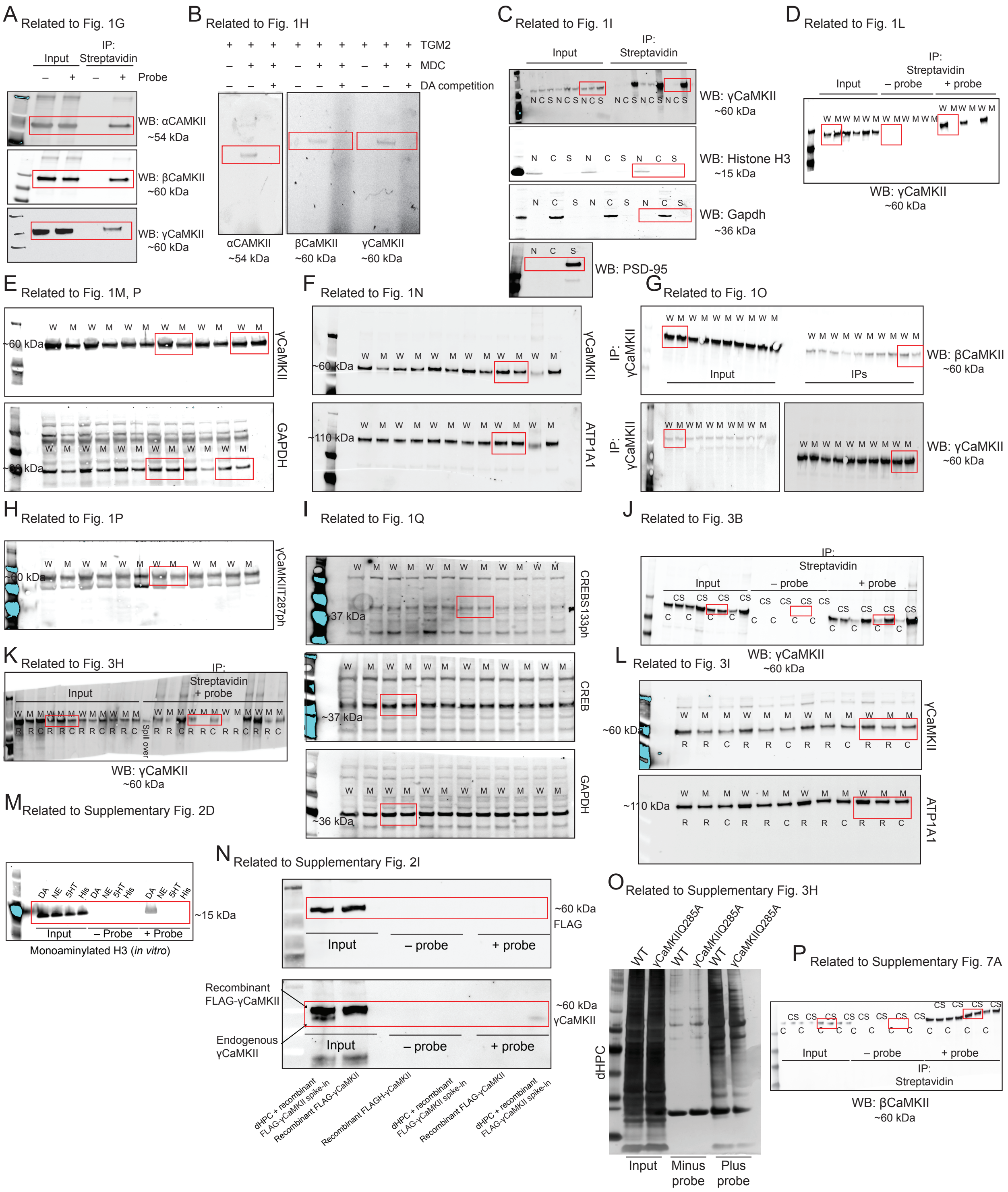

Figure S8: Uncropped blot images.
A) Uncropped blots related to **Figure 1G**.
B) Uncropped blots related to **Figure 1H**.
C) Uncropped blots related to **Figure 1I**.
D) Uncropped blots related to **Figure 1L**.

bioRxiv preprint doi: https://doi.org/10.1101/2024.09.19.613951; this version posted September 24, 2024. The copyright holder for this preprint (which was not certified by peer review) is the author/funder, who has granted bioRxiv a license to display the preprint in perpetuity. It is made available under aCC-BY 4.0 International license.

E) Uncropped blots related to **Figure 1M,P**.
F) Uncropped blots related to **Figure 1N**.
G) Uncropped blots related to **Figure 1O**.
H) Uncropped blots related to **Figure 1P**.
I) Uncropped blots related to **Figure 1Q**.
J) Uncropped blots related to **Figure 3B**.
K) Uncropped blots related to **Figure 3H**.
L) Uncropped blots related to **Figure 3I**.
M) Uncropped blots related to **Supplementary Figure 2D**.
N) Uncropped blots related to **Supplementary Figure 2I**.
O) Uncropped blots related to **Supplementary Figure 3H**.
P) Uncropped blots related to **Supplementary Figure 7A**.

Red rectangles indicate the cropped regions of blots provided in the Main and Supplementary Figures.

bioRxiv preprint doi: https://doi.org/10.1101/2024.09.19.613951; this version posted September 24, 2024. The copyright holder for this preprint (which was not certified by peer review) is the author/funder, who has granted bioRxiv a license to display the preprint in perpetuity. It is made available under aCC-BY 4.0 International license.

SUPPLEMENTARY DATA DESCRIPTIONS

Data S1. List of proteins identified by MS following Bio-CO labeling/enrichment in corticostriatal neurons plus DA treatment.

Data S2. List of proteins identified by MS following Bio-CO labeling/enrichment in corticostriatal neurons plus NE treatment.

Data S3. List of proteins identified by MS following Bio-CO labeling/enrichment in mouse VTA.

Data S4. List of proteins identified by MS following Bio-CO labeling/enrichment in mouse NAc.

Data S5. List of proteins identified by MS following Bio-CO labeling/enrichment in mouse mPFC.

Data S6. List of proteins identified by MS following Bio-CO labeling/enrichment in mouse dHPC.

Data S7. List of differentially expressed genes in dHPC comparing γCaMKIIQ285A *vs.* wildtype mice by annotated cell cluster (snRNA-seq pseudobulk analysis).

Data S8. List of genes within WGCNA modules identified in snRNA-seq dataset.
